# Polymer embedding of membrane lungs for histological investigations of intra-device clot formation

**DOI:** 10.3389/fcvm.2026.1650978

**Published:** 2026-02-04

**Authors:** Michael Kranz, Maria Stella Wagner, Daniel Pointner, Moritz Haus, Matthias Lubnow, Karla Lehle, Lars Krenkel

**Affiliations:** 1 Department of Biofluid Mechanics, Faculty of Mechanical Engineering, Technical University of Applied Sciences (OTH) Regensburg, Regensburg, Germany; 2 Regensburg Center of Biomedical Engineering, Facility of University Regensburg and Technical University of Applied Sciences (OTH) Regensburg, Regensburg, Germany; 3 Department of Cardiothoracic Surgery, University Hospital Regensburg, Regensburg, Germany; 4 Department of Internal Medicine II, University Hospital Regensburg, Regensburg, Germany

**Keywords:** clot formation, ECMO, HistoCURE 8100, histology, membrane lungs, polymer embedding, Technovit 8100

## Abstract

Extracorporeal membrane oxygenation (ECMO) is an invasive but potentially lifesaving treatment option for severe cardiac or respiratory failure. Despite its beneficial effect, coagulation-related complications, mainly due to clot formation, excessive bleeding and the accumulation of deposits in the membrane lung (ML) remain common, causing higher mortality. In this context, the formation of clots and other deposits in the ML is of particular interest. Previous histological examinations of the polymethylpentene fiber mats inside the ML could only be performed in a top view, prohibiting valid quantification and examination of the multi-layered deposits or fiber mat spanning structures. Our objective was the establishment of a polymer embedding to increase the mechanical stability of the deposits and thus enable cross-sectional microtome cutting through the ML hollow-fibers. Clinically used MLs (PLS, Getinge, Rastatt, Germany) were stabilized with a polymer resin (HistoCURE 8100). Specimens were cut out of the embedded MLs and microtome sections with a thickness of 10 µm were performed. In addition to standard histological staining with hematoxylin-eosin (HE) and Pappenheim (May-Grunwald-Giemsa), fluorescence DNA staining for nucleated cells with 4′,6-diamidino-2-phenylindole (DAPI) and SYTOX™ Green as well as immunohistochemical and immunofluorescence staining for the lysosomal enzyme myeloperoxidase (MPO) and von Willebrand factor (vWF) were established. The protocol provides a method for large volume embedding (400 mL). The cellular and extracellular deposits were securely fixed by the polymer scaffold allowing the examination of clots in MLs in native position which was not possible with conventional paraffin embedding. Multi-layered deposits and fiber mat spanning structures are no longer disrupted during specimen extraction and can now be quantified. Staining with HE, Pappenheim, DAPI, SYTOX™ Green, MPO, and vWF was successfully tested with this protocol. This method may be the foundation for new insights into the complex clotting phenomena observed in MLs.

## 1 Introduction

Extracorporeal membrane oxygenation (ECMO) is a medical device designed for treatment of transient severe lung or heart failure (1, 2). During ECMO, the patient's blood is actively drained from the patient's central venous blood vessels with a centrifugal pump and passed through a membrane lung (ML) where the blood is oxygenated and decarboxylated. Depending on the required support, the oxygenated blood is either returned to the patient's venous system (veno-venous/VV-ECMO) for pulmonary support or the arterial system (veno-arterial/VA-ECMO) for cardiac and pulmonary support (3–5).

Despite its potentially major beneficial effects, severe coagulation-associated complications during ECMO remain a major therapy limitation (6–11). Besides the development of severe bleeding, progressive clot formation in both the patient and the ECMO system is particularly problematic, as it may lead to a deterioration of the gas exchange in the ML or even a failure of the ECMO system with the need for an immediate high risk system exchange (12). As Lubnow et al. showed in their retrospective study from 2014, acute or progressive clot formation is the main reason for ECMO system exchanges (7). It is already known that elevated shear rates play a crucial role in clot formation (13–16). Elevated shear rates induce an elongation of the von Willebrand factor (vWF) in direction of the flow stress field (perpendicular to the fiber mats) (13, 15, 17, 18). This might promote clot formation despite the administration of anticoagulants in ECMO. However, the complex processes and interactions between the patient's blood and the ML are not yet sufficiently understood and require further investigation, focusing on the deposits on the ML fibers in particular.

There are a variety of ML designs currently at use in clinical practice. This work focuses on Permanent Life Support (PLS) MLs (Getinge, Rastatt, Germany). These are structured in three separate compartments: The blood compartment in which the patient's blood flows through and in which clots deposition can be observed. The gas compartment, through which a gas mixture with a high O_2_ and low CO_2_ content flows, and the heat exchanging compartment in which tempered water flows.

The blood and the gas compartments are separated by semipermeable polymethylpentene (PMP) hollow-fibers, woven to fiber mats using warp threads. Blood flows outside the PMP fibers and O_2_ flows within. Gas exchange occurs due to a concentration gradient between blood and gas compartment. In contrast, the blood and the heat exchanging compartments are separated by non-permeable thermoplastic polyurethane (TPU) hollow-fibers. With tempered water inside the hollow-fibers, the blood temperature is controlled without any direct contact between the compartments. During ECMO, blood is directed through a stack of 45 alternating PMP (*n* = 23) and TPU (*n* = 22) fiber mats. After passing the dividing wall, a coarse polycarbonate grid, the blood passes a second fiber mat stack consisting of 74 PMP fiber mats before being returned into the patient's vascular system.

**Figure 1:**
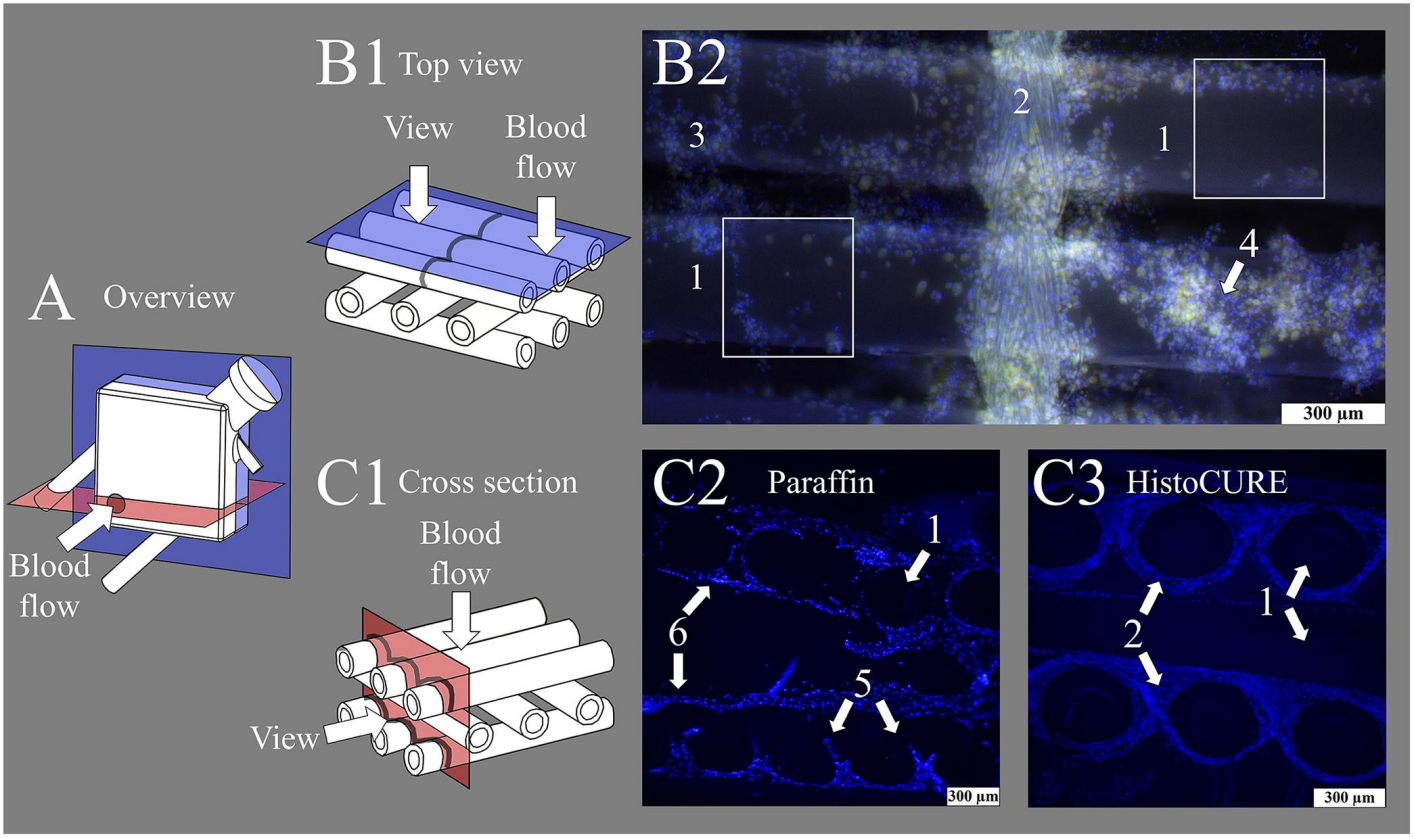
Display of fiber mats captured with fluorescence microscope at 100-fold magnification. **(A)** Overview image of membrane lung (ML) structure with viewing planes; gray circle indicating position of inlet port. (**B1**) Schematic drawing of state-of-the-art top view investigations of fiber mats. (**B2**) Nuclei stained with DAPI (blue); red blood cells (RBCs) visible due to autofluorescence (yellow). Display of (1) hollow-fibers woven together with a (2) warp thread. Cellular deposits heterogeneously distributed, especially at fiber mat (3) crossing points or in (4) multi-layered clot structures. White squares indicate areas that are analyzable with the existing methodology using top view microscopy. (**C1**) Schematic drawing of cross-sectional view of fiber mat stack. Cross-sectional view of fiber mat stack embedded with (**C2**) conventional paraffin and (**C3**) HistoCURE 8100 polymer solution. (**C2**) Showing (5) disrupted clot structures and (6) displaced hollow-fibers. (**C3**) (1) Hollow-fibers are securely fixed in native position; polymer embedding also allows sectioning of the intertwining (2) warp threads.

To date, deposits on fibers of clinically used MLs were mostly examined through manual extraction of single fiber mats from the stack and subsequent fluorescence staining, which only allowed investigations from a top view perspective (19, 20). However, multi-layered deposits or blood clots could not be quantified in this manner, as the lack in depth information impeded discrimination of individual cell layers (Figure 1). Furthermore, the forceful removal of the fiber mats from the stack resulted in a disruption of structures spanning between the fiber mats, losing them for the investigation.

However, as we expect those fiber mat spanning deposits and clots to be found frequently in MLs from previous investigations (7, 12, 20–27), a method was required to enable cross-sectional examination of the ML hollow-fibers, their deposits and clot structures. We expect the presence of fiber mat spanning deposits in clinically used MLs. However, existing investigation methods do not allow any statements to be made in this regard. It therefore requires standardized microtome sections through clot structures and ML hollow-fibers. In preliminary tests of native (non-embedded) ML fibers, it became evident that the delicate hollow-fibers and deposits were mainly disrupted during microtome sectioning which did not allow any reproducible histological investigation. Hence, the objective was to increase the mechanical stability of the ML and the contained clots to enable standardized microtome sectioning and subsequent staining.

First approaches with stabilizing clots and hollow-fibers were conducted by Steiger et al. (19). They showed exemplarily the use of paraffin to stabilize ML fibers (19). However, paraffin did not allow reproducible embedding of larger specimens which did not permit systematic and large-scale ML investigations (Figure 1). Moreover, the different material hardnesses (ML fibers vs. paraffin) significantly restricted the sectioning. Moreover, paraffin did not allow the filling of the hollow-fibers' interior due to its relatively high viscosity. However, by stabilizing not just the outside but also the inside of the hollow-fibers, with a scaffold of similar hardnesses as the hollow-fibers, a composite of high sectioning quality should be achieved. To do so, polymer embedding of the entire ML was chosen.

One of the most commonly used polymers in histology is Technovit® 9100. Its main advantage is the capability of deplastification and thus the accessibility of antigens for immunohistological investigations. However, in the early development stages of this protocol, we observed interactions between the polymer and the ML hollow-fibers leading to a fiber elongation of approximately 10%. Furthermore, xylene was needed for the preparation process, which resolved the plasticizers and thus destroyed the ML housing. Ultimately, a protocol using HistoCURE 8100 (formally known as Technovit® 8100) was developed to effectively circumvent these issues.

This work outlines the protocol used for the polymer embedding of MLs and the systematic extraction and microtome sectioning of the specimens. It also demonstrates various staining strategies, potential pitfalls and errors regarding protocol execution, as well as their prevention. This method marks an important step towards a better accessibility of the complex multilayered structures found in MLs. The gained knowledge can be the foundation for optimizing blood-transporting medical devices.

## 2 Materials and equipment

The Tables 1, 2 contain the most important equipment, materials, and reagents required for embedding and staining.

**Table 1:** List of required equipment.

No.	Item	Qty	Description	Supplier
Polymer embedding and specimen extraction				
1	Permanent Life Support (PLS)	1	Embedded and investigated ML	Getinge, Rastatt, Germany
2	Technovit® embedding molds with hexagonal inserts	18	Embedding molds for specimen extraction	Morphisto, Offenbach, Germany
3	Micro V G222E O_2_ monitor	1	Monitoring O_2_ during CO_2_ use	GfG, Dortmund, Germany
4	MH 20 magnetic stirrer	1	Stirring of polymer solution	Carl Roth, Karlsruhe, Germany
5	Testo 835-T1 infrared thermometer	1	Monitoring polymerization temperature	Testo, Titisee-Neustadt, Germany
6	Dremel 4000	1	Cutting off connector ports of ML	Dremel, Racine, WI, USA
7	TC-SB 305 U band saw	1	Cutting out specimens from embedded ML	Einhell, Landau, Germany
Microtome sectioning				
8	HM355S microtome	1	Microtome sectioning of embedded ML specimens	Thermo Fisher, Waltham, MA, USA
9	R16D Tungsten-carbide D-knife 16 cm	1		Gigatome, Roggenburg, Germany
10	Cylindrical microtome mounting with fixation of 25 mm diameter specimens	1		OTH Regensburg, Regensburg, Germany
11	Superfrost Ultra Plus® glass slides	1 pck	Glass slides	Thermo Fisher, Waltham, MA, USA
Image acquisition and processing				
12	DMi8 inverse microscope with a LED 5 light source	1	Imaging of sections from embedded ML	Leica Microsystems, Wetzlar, Germany
13	K3C RGB camera	1	Transmitting light microscopy	
14	K5 grayscale camera	1	Fluorescence microscopy	
15	DFT51011 filter cube (420–450 nm, 506–532 nm, 578–610 nm, 666–724 nm)	1	Optical filter for fluorescence microscopy	
16	DFT5 filter wheel (420–460 nm, 500–540 nm, 565–615 nm, 662–738 nm)	1		
17	LAS X 3.10 software	1	Control software for DMi8 microscope	
18	Adobe Photoshop CS5 64 bit	1	Imaging software to reduce background	Adobe, San José, CA, USA

**Table 2:** List of required reagents and solutions.

No.	Reagent	Art. No./supplier
Embedding polymers		
19	HistoCURE 8100 Set 500 mL (2 sets)	12226.K0500, Morphisto, Offenbach, Germany
20	Technovit® 3040 set	12226.K0180, Morphisto, Offenbach, Germany
Fixative and buffer solutions		
21	NaCl 0.9%	1310181, Fresenius-Kabi, Bad Homburg, Germany
22	Paraformaldehyde	104005, Merck, Darmstadt, Germany
23	Methanol	4627, Carl Roth, Karlsruhe, Germany
24	NaOH flakes	LC4994.1, LaboChem, Ruse, Bulgaria
25	Na_2_HPO_4_	106579, Merck, Darmstadt, Germany
26	KCl	HN02.2, Carl Roth, Karlsruhe, Germany
27	KH_2_PO_4_	104873, Merck, Darmstadt, Germany
28	NaCl	106400, Merck, Darmstadt, Germany
29	Tris-Base	T1503–1 kg, Sigma-Aldrich, Steinheim, Germany
30	Tris-HCl	9090.2, Carl Roth, Karlsruhe, Germany
31	Bovine serum albumin (BSA), Frakt. V	8076.2, Carl Roth, Karlsruhe, Germany
32	Gelantin from cold water fish skin	G-7765, Sigma-Aldrich, Steinheim, Germany
33	Normal goat serum (NGS)	G-9023, Sigma-Aldrich, Steinheim, Germany
34	Normal donkey serum (NDS)	D-9663, Sigma-Aldrich, Steinheim, Germany
Histological staining solutions		
35	Mayeŕs hemalum solution	1.09249.0500, Sigma-Aldrich, Steinheim, Germany
36	Eosin	15935, Merck, Darmstadt, Germany
37	May-Grunwald stain	63595, Sigma-Aldrich, Steinheim, Germany
38	Giemsa stain	48900, Sigma-Aldrich, Steinheim, Germany
Antibodies		
39	Rabbit, anti human, anti-Myeloperoxidase (MPO)	A0398, DAKO Cytomation, Wiesentheid, Germany
40	Rabbit, anti human, anti-von Willebrand factor (vWF)	A0082, DAKO Cytomation, Wiesentheid, Germany
41	Rabbit, anti human, isotype control antibody	31235, Invitrogen, Waltham, MA, USA
42	Goat, anti rabbit, biotinylated antibody	BA-1000, Vector Laboratories, Newark, CA, USA
43	Donkey, anti rabbit AlexaFluor 647	A-31573, Invitrogen, Waltham, MA, USA
Agents and stains for immunhistochemistry (IHC) and for immunohistofluorescence (IHF)		
44	Vectastain® Elite® ABC Kit, Peroxidase (Standard)	PK-6100, Vector Laboratories, Newark, CA, USA
45	Histogreen	E109, Linaris/Biozol Diagnostica, Eching, Germany
46	DAB-Liquid	K3468, Dako/Agilent, Santa Clara, CA, USA
47	SYTOX™ Green	S-7020, Thermo Fisher, Waltham, MA, USA
Mounting agents		
48	Entellan^TM^	1.07961.0500, Merck, Darmstadt, Germany
49	VectaMount® AQ	H-5501, Vector Laboratories, Newark, CA, USA
50	Flouromount-G™	00–4958-02, Thermo Fisher, Waltham, MA, USA
51	Flouromount-G™ DAPI	00-4959-52, Thermo Fisher, Waltham, MA, USA
Miscellaneous reagents		
52	Ethanol absolute	107017, Merck, Darmstadt, Germany
53	HCl	1.00317.0510, Merck, Darmstadt, Germany
54	Acetic acid 100%	1.00063.1011, Merck, Darmstadt, Germany
55	Xylene, (mixture of isomers) ≥98%	28973.363, VWR, Radnor, PA, USA
56	H_2_O_2_	386790, Merck, Darmstadt, Germany
57	Trypsin-EDTA	T4174, Sigma-Aldrich, Steinheim, Germany
58	CaCl_2_ (1M)	21114, Honeywell Fluka, Sleeze, Germany
59	Citric buffer (pH 6)	ZUC 028-500, Zytomed, Berlin, Germany
60	Glycerol >99%	SHBL3980, Sigma-Aldrich, Steinheim, Germany

## 3 Methods

**Figure 2:**
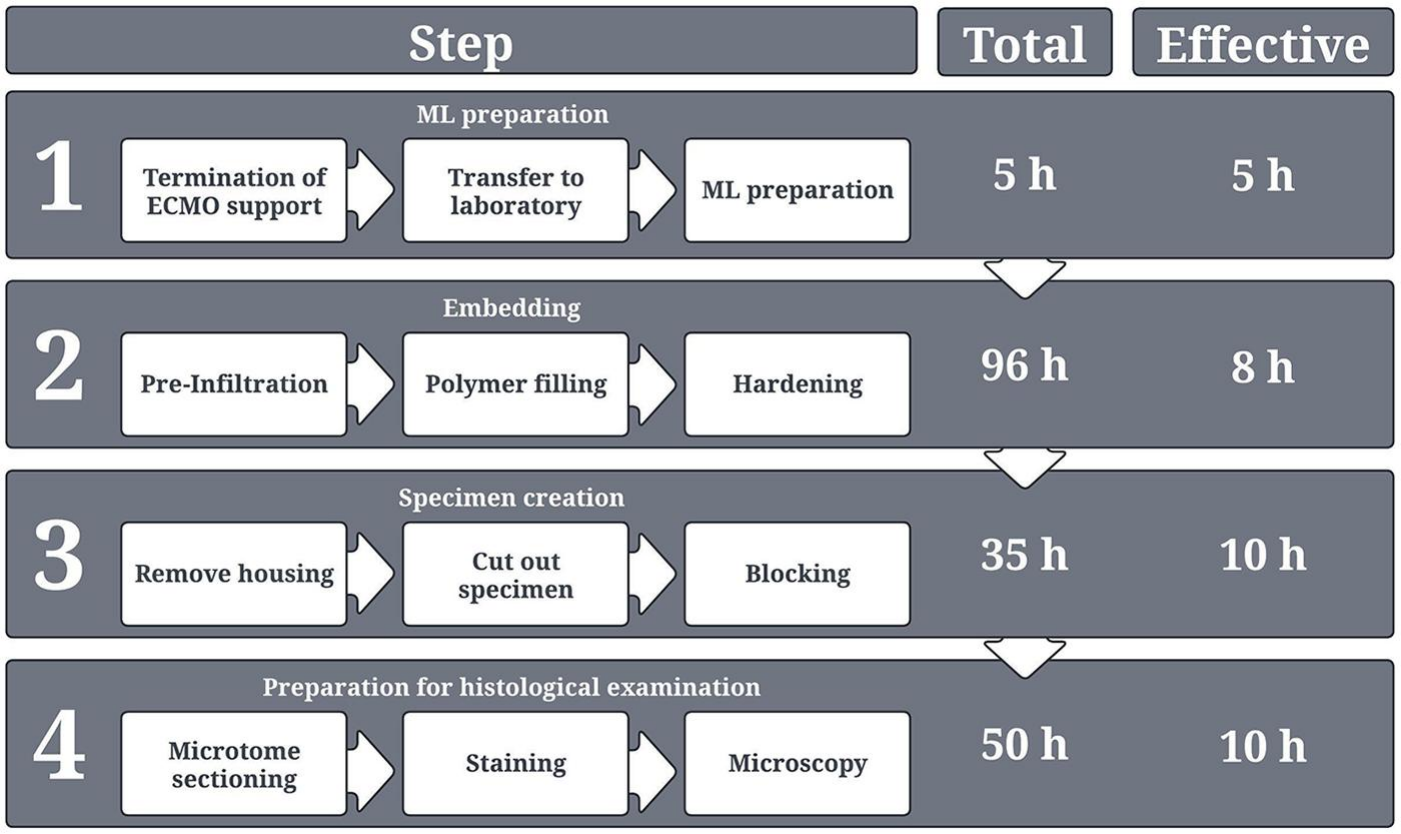
Overview of the entire process from ML collection in the hospital up to histological examination. The respective duration for each process step is provided. A distinction is made between “total time,” which describes the total duration of each process step, including waiting times, hardening times, or application times for stains etc., and “effective time,” which in turn describes only the actual working time for two persons. The depicted times present the sum of the duration of each individual sub-processes of the respective process step.

Clinically used end-of-therapy MLs (Table 1 item #1) were embedded following our developed embedding protocol. In summary: The MLs were collected after ECMO therapy and filled with HistoCURE 8100 (Table 2 item #19). After the polymer was fully hardened, the ML housing was opened. Regions of interest (ROIs) were sawed out and blocked in embedding molds (Table 1 item #2) using HistoCURE 8100 for embedding and Technovit® 3040 (Table 2 item #20) for specimen block creation. Microtome sections of a 10 µm thickness were performed, followed by histological staining with various agents and protocols. The entire embedding process took about 96 h in total for one ML. The effective working time of the embedding process is 8 h for two people. The entire process from retrieving the ML after clinical use up to microscopic image acquisition is described in detail below with a visual overview given in Figure 2. In addition, a flow chart of the procedure with a more detailed description is provided in the Supplementary Files.

Investigation of clinically used MLs from terminated ECMO support was prior approved by the University of Regensburg Ethics Committee (vote no. 20-2051-104).

### 3.1 ML preparation after ECMO use

All MLs that were required for the establishment of this protocol were collected at the University Hospital Regensburg after termination of ECMO support. To remove residual, non-adherent blood components, the MLs were rinsed with 10 L of isotonic saline solution (Table 2 item #20). The adherent deposits were fixed with formol solution [4% m/v paraformaldehyde, in 0.1 M phosphate buffered saline (PBS: 1.15% m/v Na_2_HPO_4_, 0.20% m/v KCl, 0.20% m/v KH_2_PO_4_, 8% m/v NaCl in distilled water), pH 7.3 + 10% v/v methanol, 1 L, Table 2 items #22–28]. After fixation, the MLs were stored at 4 °C until further processing (20, 28, 29).

### 3.2 Estimation of clot burden

**Figure 3:**
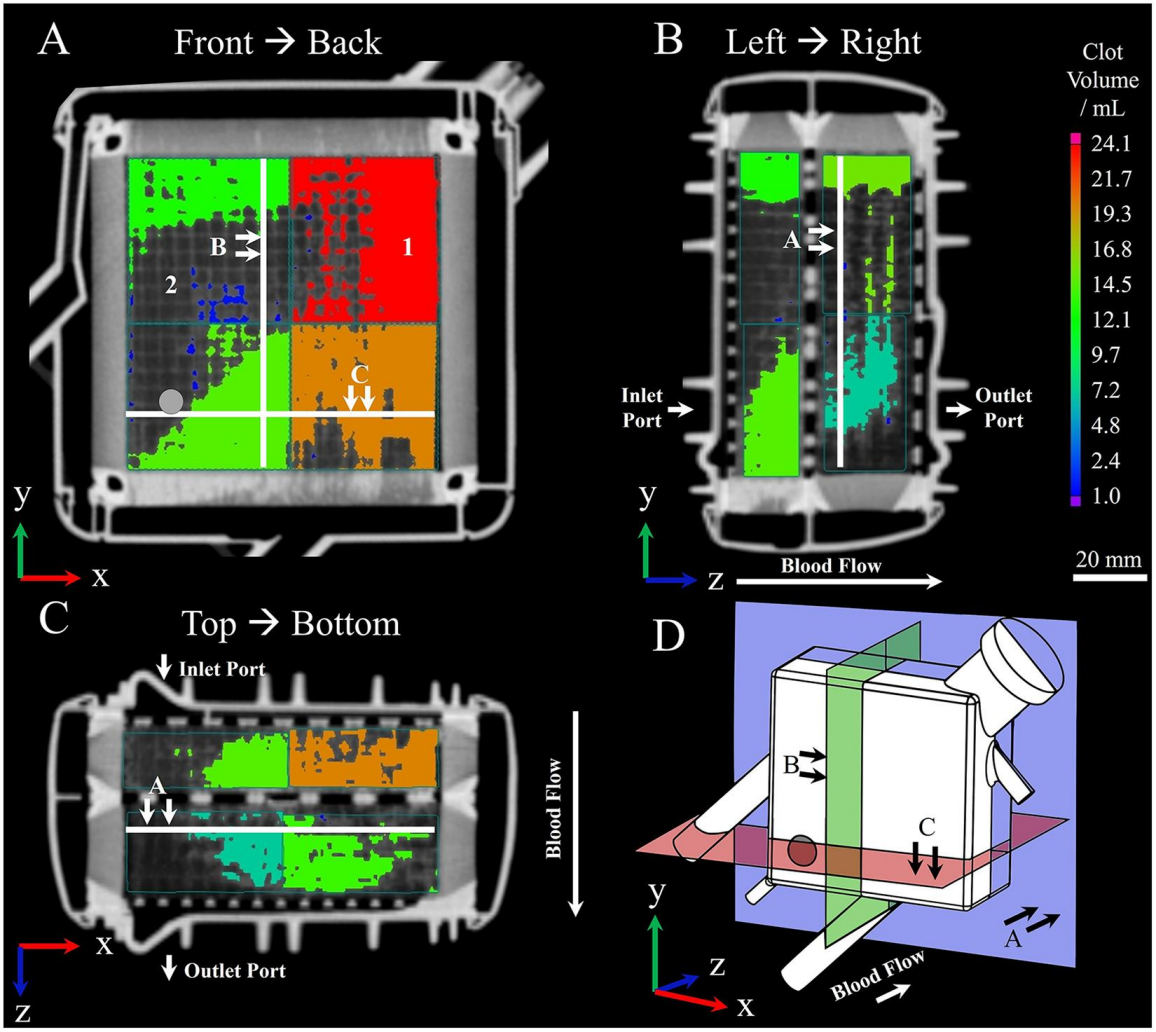
Visualization of (1) clotted and (2) clot-free areas within MLs prior to embedding using clinical multidetector computed tomography; procedure according to Wagner et al. (20). Clot depositions are shown in color. Wagner et al. (20) virtually divided the ML into eight sectors and calculated the contained clot volume within by applying a threshold-based algorithm. The resulting clot volume is color-coded and corresponds to the color bar located on the right-hand side. **(A)** Front to back perspective; gray circle indicating position of inlet port. **(B)** Left to right perspective. **(C)** Top to bottom perspective. **(D)** Three-dimensional representation of displayed planes.

This intermediate step is not strictly necessary for embedding but it allows a quick estimation of the clot burden of the ML in advance. Clot visualization utilizing clinically used multidetector computed tomography (MDCT) was previously described (20, 26, 30, 31). With this imaging modality, ROIs can be determined which may be later relevant for histological investigation. We recommend performing MDCT scans prior to embedding, due to the difficult discrimination of polymer and hollow-fibers, as preliminary tests indicated. As an example, Figure 3 depicts the clot volume at a representative position within an ML.

### 3.3 ML preparation for embedding

**Figure 4:**
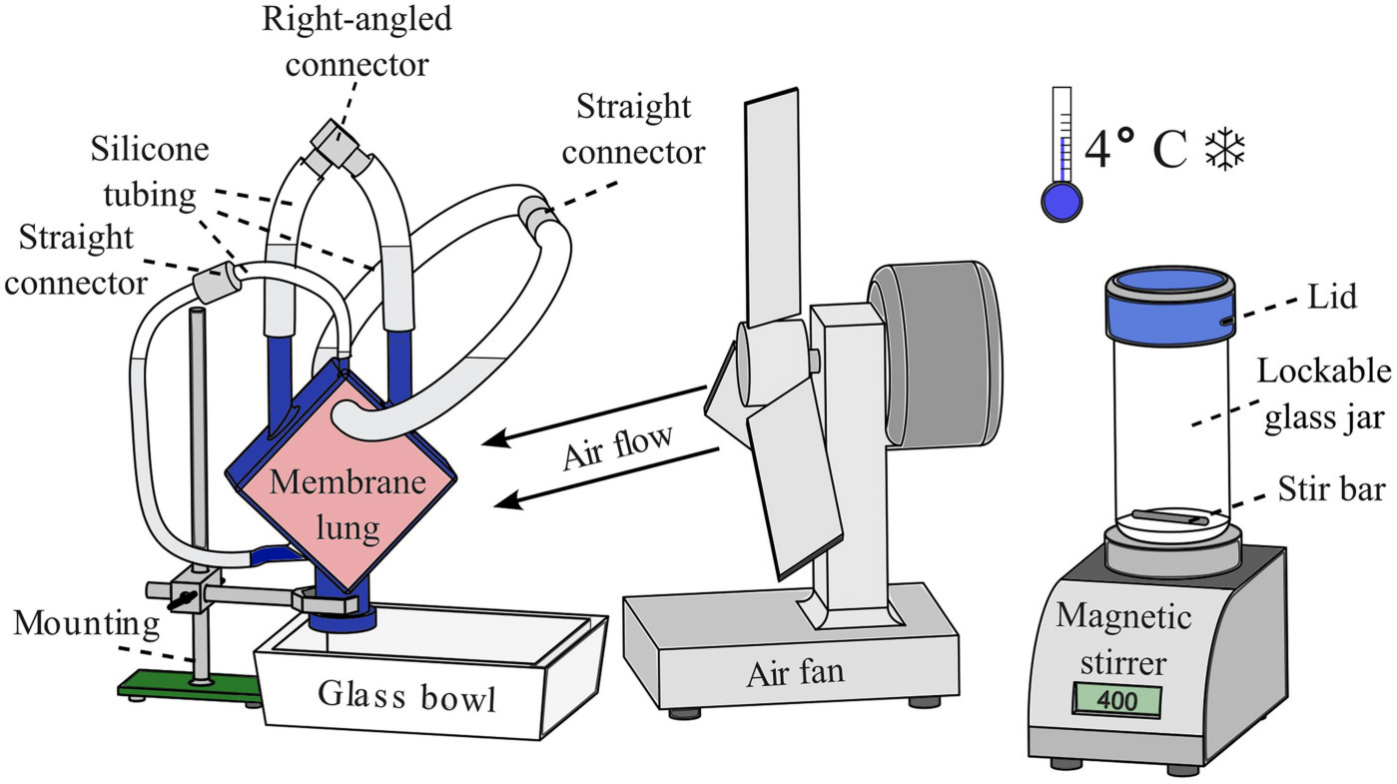
Setup for the embedding of MLs. Left: ML fixed to a mounting with the connectors facing upwards to let residual air rise into the silicone tubes; a glass bowl underneath captures the polymer in case of leakage. Middle: an air fan is used to improve the removal of the developing heat during polymerization. Right: a magnetic stirrer with a lockable 1 L glass jar is used to mix the HistoCURE 8100 base solution with the hardeners at 400 RPM; the entire procedure is conducted at an ambient temperature of 4 °C.

To avoid excessive heat development due to the exothermic polymerization, the ML as well as all required components and tools were tempered to 4 °C. Furthermore, draining of the fixative solution, preparing the ML, embedding, and hardening took place in a well-ventilated room tempered to 4 °C. After removing the formol fixative, silicone tubes with a length of 25 cm (diameter 10 mm for blood and heat exchanging compartment and 6 mm for gas compartment) were attached to the ML ports connecting the inlet and outlet port of each compartment. This facilitated the further preparation process (Figure 4). Cable ties secured the tubes in place and prevented leaking. The ML was rinsed with 2 L of isotonic saline solution (Table 2 item #21) through the blood inlet, the gas inlet, and the water inlet. Proper tightness of the tube connections should be checked at this stage. Subsequently, the ML was dried with compressed air until no water residues were visible. Pivoting the ML helped drain the water during air drying. Then, the ML was filled with 500 mL ethanol (Table 2 item #52) using a 100 mL syringe. The multi-step dehydration according to the manufacturer's protocol was dispensed to prevent clot structures from being rinsed out. Instead, only a single step using 99.97% ethanol was conducted with draining after 7 min. As O_2_ inhibits the polymer hardening reaction, every compartment of the ML was flushed with CO_2_ for 30 s. For safety reasons, an O_2_ monitoring device (Table 1 item #3) is recommended to be used during this step.

### 3.4 Embedding

The MLs were embedded in liquid HistoCURE 8100 polymer solution, a glycol methacrylate acetate (32) which was developed in particular to increase the mechanical stability of biological specimens for microtome sectioning. Another advantage of HistoCURE 8100 was its capability to conduct immunohistochemical analysis (32, 33). The embedding procedure was based on the manufacturer's guidelines. As previous works only described the application of HistoCURE 8100 in specimens up to 2 cm^3^ (34), the manufacturer's protocol had to be adapted to fill the required volume of 400 mL with polymer. The embedding setup is depicted in Figure 4.

300 mL of the HistoCURE 8100 base solution and 1.8 g of hardener 1 were filled in a 1 L lockable glass jar. The mixture was stirred for 30 min at 400 RPM using a magnetic stirrer (Table 1 item #4) and a 2 cm long stir bar. The mixed solution was filled inside the blood compartment to pre-infiltrate the deposed clots for 72 h allowing a better binding with the later used hardening solution. After pre-infiltration, 500 mL of HistoCURE 8100 base solution and 3 g of hardener 1 were stirred for 30 min as described above. 34 mL of hardener 2 were added to the mixture using a 20 mL syringe. CO_2_ was flushed inside the jar for 10 s to remove any O_2_ before the lid was closed. The substances were stirred for about 7 min until a green discoloration was visible. It was filled inside the ML through the connected silicone tubing using a sterile 100 mL syringe pushing out the pre-infiltration solution at the opposite side. The compartments and the connected tubing were filled up to 50 mm above the ML connectors to compensate for volume loss of the polymer while hardening. Before sealing, CO_2_ was flushed into the silicone tubes to avoid any contact with O_2_. Heat removal during the exothermic polymerization reaction was facilitated by a cooling fan. Until the end of the main reaction after 4 h, regular temperature monitoring of the front and back of the ML was performed with an infrared thermometer (Table 1 item #5). In case of non-uniform temperature rise, the fan position should be adjusted. Maximum temperature (about 30 °C at the ML housing) was observed after about 3 h and after 24 h in the cooling room at ambient temperature of 4 °C, the ML was fully hardened, and specimens were cut out.

### 3.5 Creation of specimen

**Figure 5:**
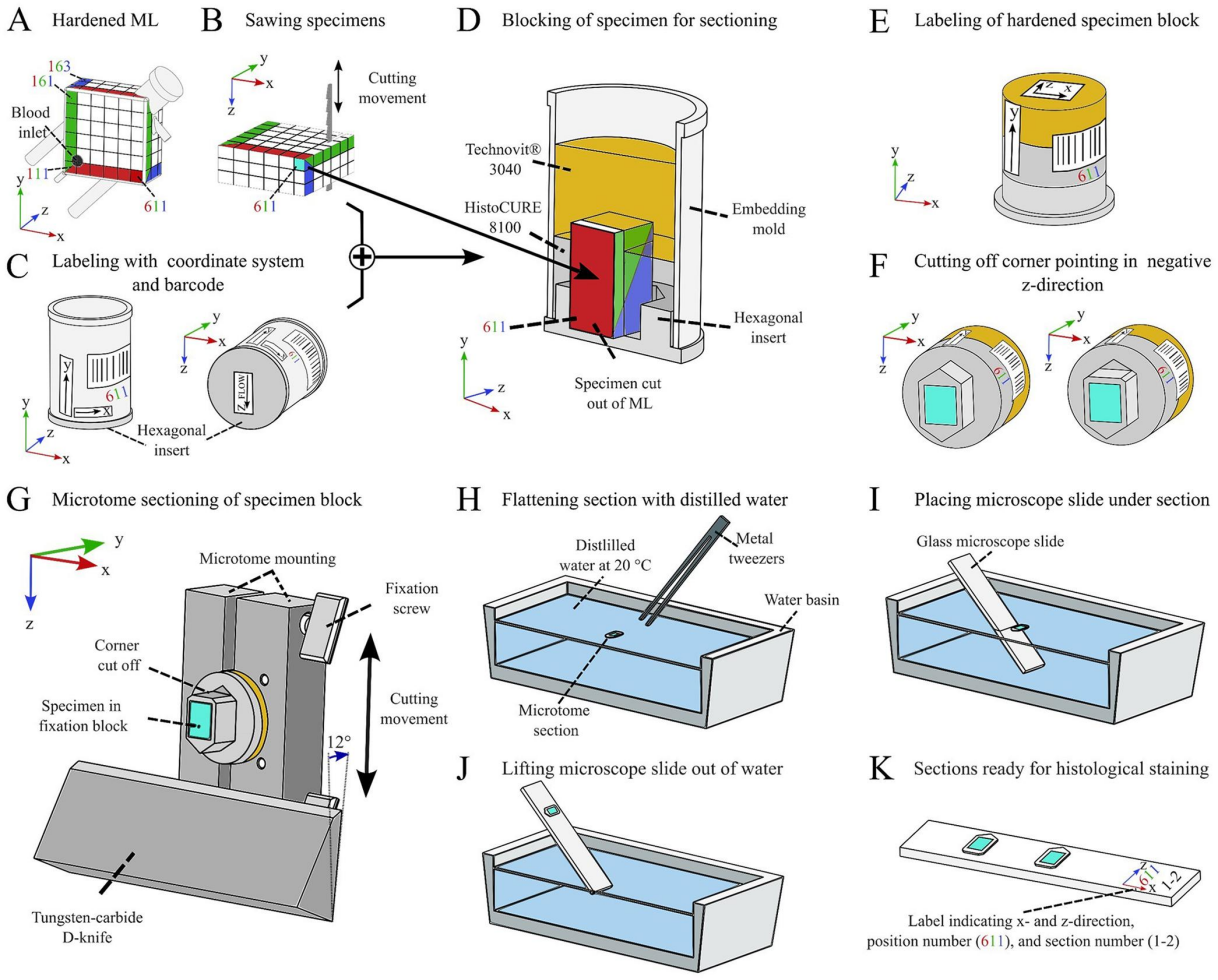
Process of systematic specimen extraction from the embedded ML. **(A)** Assigning a coordinate system for maintaining orientation of each specimen. **(B)** Sawing the individual specimens with a band saw. **(C)** Labeling the embedding mold with coordinate markers. **(D)** Embedding the specimen in a block using a combination of HistoCURE 8100 and Technovit® 3040. **(E)** Removing specimen from embedding mold; leaving hexagonal insert attached to specimen to maintain z-direction. **(F)** Cutting off corner pointing in negative z-direction to maintain z-direction of microtome sections. **(G)** Performing microtome sections with a cylindrical mounting and a tungsten-carbide D-knife; lower corner of specimen is pointing in positive z-direction; transferring the section to a water basin with metal tweezers. **(H)** Flattening the section by carefully placing it on the water surface of tempered (20 °C) distilled water. **(I)** Placing a glass microscope slide diagonally under the flattened section. **(J)** Lifting up the glass slide; the section adheres to the slide. **(K)** Multiple sections can be placed on one slide for later histological staining.

To create specimens from the ML, the lateral housing had to be removed. First, all connectors were cut off with a Dremel 4000 (Table 1 item #6). This facilitated the later removal of the outer housing with a band saw (Table 1 item #7). Depending on later application, it may be important to maintain the initial orientation of the specimen. This was achieved by using a coordinate system which assigned location numbers for every specimen (Figure 5A). To prevent any contamination of the hardened fiber mat stack, it was wrapped in adhesive tape. A printed cutting template (see Supplementary Files) was attached on top to facilitate the sawing process. The specimens were cut out according to Figures 5A,B. After the surrounding tape was removed, the specimens were immediately placed into a cylindrical embedding mold with a hexagonal insert (Figures 5C,D, Table 1 item #2). Arrows were attached to the embedding molds to visualize the coordinate axes (Figure 5C). Placing the cut-out specimens in the exact orientation indicated on the embedding mold is crucial, as an error at this point may not be detectable later. All labels required for specimen creation can be found in the Supplementary Files.

The location number was also integrated into a barcode that was labeled on each embedding mold to identify all individual specimens (Figure 5C) and link them to a database documenting specific specimen properties (e.g., date of embedding, clot burden, quality of specimen, cutting behavior etc.). The embedded specimens were placed in another embedding mold and were encapsulated with additional polymer to form a specimen block (Figure 5D), which is later cut using a microtome (Section 3.6). This process is referred to as “blocking”. It is not necessary to saw out and block out all 108 specimens immediately after embedding. Instead, specimens that have not been sawed out can be stored at 4 °C and blocked later. We found that even after more than one and a half years after embedding, the samples could still be processed and analyzed.

In this step, 18 specimens were processed simultaneously, as this appeared to be optimal quantity of specimens to handle at once. The embedding molds were positioned in a shallow, sealable glass container with the cut-out specimens (Figure 5D) placed in the center of the hexagonal mold insert. The glass container was then placed in a cooling room at 4 °C for 3 h before block preparation. The following blocking procedure was performed similarly to the ML embedding at 4 °C ambient temperature (specimens and material previously tempered to 4 °C). 5 mL of HistoCURE 8100 hardening solution (in total: 100 mL base solution + 1 package of hardener 1 + 7 mL of hardener 2, processed as mentioned before; 18 specimens require 90 mL and 10 mL are included as a reserve) were poured in each embedding mold. Specimen displacement due to polymer pouring should be corrected. Attention must be paid here to prevent the formation of air bubbles under or next to the cut-out specimen. In this case, the block would not harden sufficiently. CO_2_ was again used in the HistoCURE 8100 hardening process for O_2_ removal by flushing the container for 30 s and sealing it with a lid. After 24 h, the HistoCURE 8100 had fully hardened. The embedding molds were removed from the container and placed in a laboratory fume hood at room temperature (RT, 20 °C).

The following steps were all conducted at RT. After reaching RT, each mold was filled with Technovit® 3040 hardening solution to just before the upper edge of the mold. This solution was made with a mixing ratio of 2 parts of powder and 1 part of liquid (10 mL powder and 5 mL liquid per embedding mold, 180 mL powder and 90 mL liquid in total). These components were filled into a disposable cup and were stirred with a glass bar until no lumps were visible (about 10 s) before pouring into the embedding molds. After 30 min, the Technovit® 3040 was completely hardened. The specimen blocks were removed from the molds and labeled according to the labeling on the outside of the mold (Figure 5E). Then, the corresponding barcode sticker was placed on the side of the specimen (Figure 5E). To recognize the z-direction after microtome sectioning, the corner of the specimen pointing in the negative z-direction was removed with a handsaw (Figure 5F). The remaining corner thus points like an arrow in the z-direction (Figure 5F). The specimens were stored at RT until microtome sectioning. We recommend storing them in a well-ventilated room since within the first weeks after blocking, the polymer emits chemical vapors.

Using a combination of two polymers for specimen blocking showed distinct advantages: The use of HistoCURE 8100 in the cutting area of the specimens improved their cutting behavior, due to its lower hardness. Technovit® 3040, on the other hand, showed higher hardness, it was easier to handle, and cheaper to procure. The area of the specimen block, which was later clamped in the microtome mounting, was therefore processed with Technovit® 3040. Furthermore, pouring two thinner layers of HistoCURE 8100 and Technovit® 3040 instead of one thicker HistoCURE 8100 layer, improved heat removal and therefore reduced the formation of air bubbles within the polymer. These would negatively affect the sectioning quality.

### 3.6 Microtome sectioning and specimen placement on microscope slides

**Figure 6:**
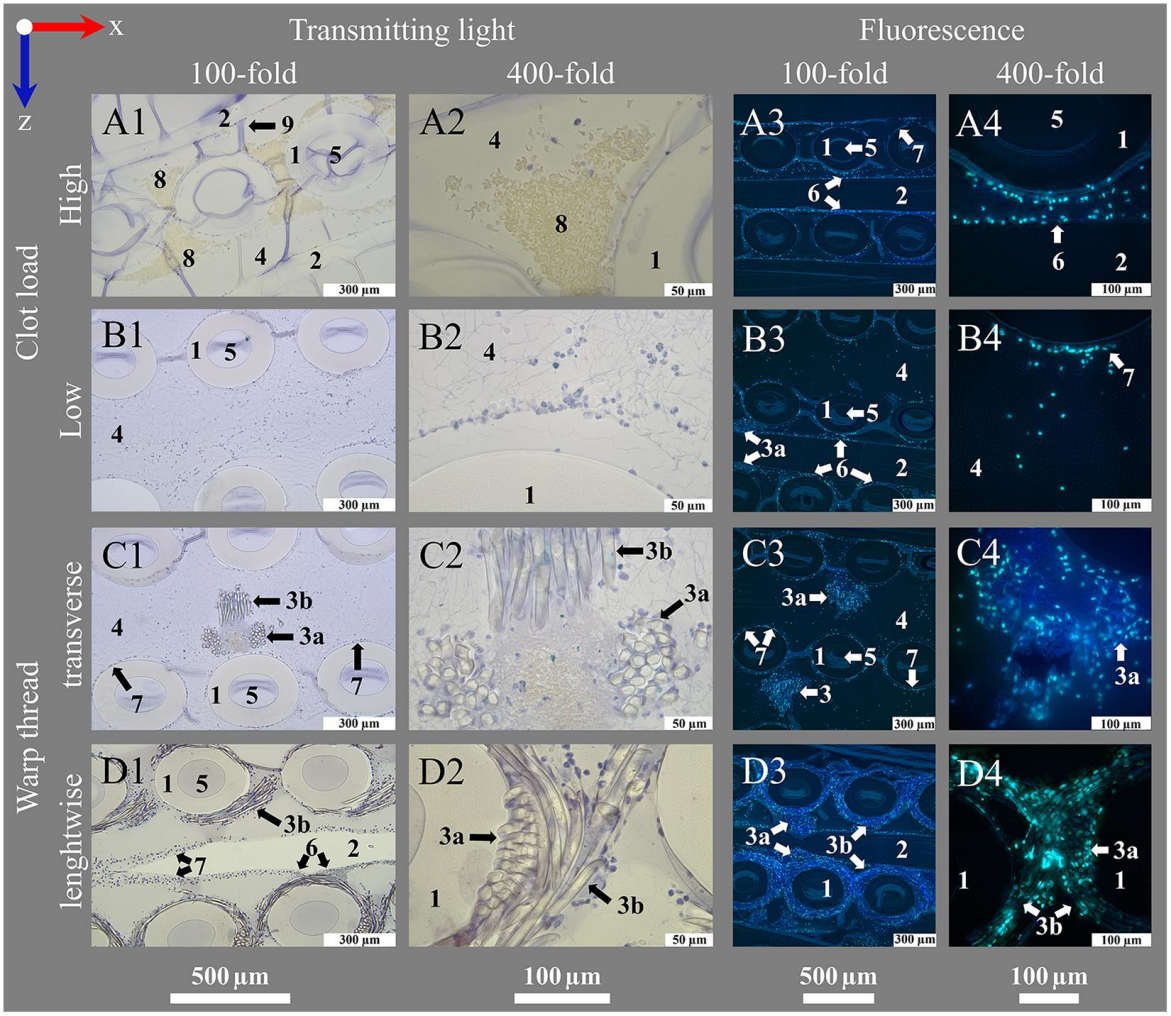
Introduction of important structures within an ML visualized by transmitting light and fluorescence microscopy. An ML consists of PMP fiber mats stacked at 90 °. After polymer embedding, sections consist of (1) cross-sections and (2) longitudinal sections of PMP fibers, visible in the (**A1–D1**, **A3–D3**) overview (100-fold magnification). Fibers are woven into mats by (3) a connecting warp thread. The (4) blood compartment as well as (5) the inside of the fibers are filled with HistoCURE 8100. Especially (6) crossing points of two PMP fibers, (7) the surface of PMP fibers and warp threads [(3a) cross-section, (3b) longitudinal section of strands] are prone to attachment of nuclear deposits and formation of (8) clots. Blood flow (blue arrow) in downwards direction in the images. (**A2–D2**, **A4–D4**) Detailed images (400-fold magnification) allow a closer look at the biological deposits. Blood flow within the ML from up to down in the sections. (**A1–D2**) Transmitting light microscopy and (**A3–D4**) fluorescence microscopy images show comparable structures in each row. Biological deposits in the blood compartment are stained with (**A1–D2**) Hemalum (nucleated cells blue) and anti-Myeloperoxidase (MPO green, visualized with Histogreen) and (**A3–D4**) DAPI and SYTOX™ Green for visualization of nuclei (DAPI blue; SYTOX™ Green green) with fluorescence phase contrast (turquoise) for visualization of hollow-fibers and warp threads (only overlay images depicted, separate images of all channels in Supplementary Figures S1, S2. Except **A1**, **A2** (5 µm), all sections are cut with a thickness of 10 µm, where (9) folds are created. **(A)** Clots within the ML; (**A1**) clots are located between the cross-sections of PMP fibers; (**A2**) higher magnification allows the differentiation between red blood cells (native yellow) and nucleated cells (hemalum blue); (**A3**) visualization of a clotted region through fluorescence; (**A4**) a crossing point. **(B)** Clot-free region of the ML; (**B1**) mostly single cells attached to the PMP fibers or stretching into the blood compartment; two fiber mats are cross-sectioned, the sample is located between two fibers of the longitudinal mat (only blood compartment visible, longitudinal fibers not visible); (**B2**) nuclear deposits in the blood compartment close to a PMP fiber cross-section; (**B3**) three cross-sectioned mats and one longitudinal sectioned mat, instead of the second longitudinal mat, blood compartment is visible; (**B4**) nuclear deposits stretching in the interspace between the fibers. **(C)** Cross-section of a warp thread; (**C1**) two cross-sectioned fiber mats and a warp thread, which belongs to the longitudinal mat, that is not cut in this section; (**C2**) knot of the warp thread with high load of biological deposits in the center; (**C3**) two cross-sectioned fiber mats with two warp thread knots, both belonging to the in between longitudinal mat; (**C4**) nuclear deposits mainly attached to the strands of the warp thread. **(D)** Longitudinal cut warp thread; (**D1**) two cross-sectioned fiber mats surrounded by their warp thread, between them a longitudinal cut fiber of the alternating mat; (**D2**) different strand directions of the longitudinal warp threads around two fibers visible; (**D3**) two longitudinal warp threads around the cross-sectioned fiber mats and a longitudinal cut fiber of the alternating mat, also with a warp thread; (**D4**) longitudinal and cross-sectioned strands of a warp thread around two fibers.

A rotary microtome (Table 1 item #8) equipped with a tungsten-carbide D-knife (Table 1 item #9) was used for sectioning. Sectioning was performed with an in-house designed microtome mounting featuring a fixture (Table 1 item #10) for cylindrical specimen blocks with a diameter of 25 mm (Figure 5G). The knife was tilted 12 ° towards the microtome mounting. Before cutting analyzable sections, the upper layer of the specimen block was trimmed until the hollow-fibers were exposed. This created an even surface for the actual sections. In this step, great care had to be taken to prevent breaking the blocks. Best trimming results were achieved with a section thickness of 4 µm. Trimming and specimen sectioning were carried out using the microtome's manual drive with a preferably high cutting speed. The actual specimen sectioning was carried out with 5 µm and 10 µm thickness, whereby the latter one showed better handling in subsequent histological staining. After sectioning, the section was carefully picked up with pointed metal tweezers and placed on the surface of a water basin filled with distilled water (water at RT, Figure 5H). The section then rolled out flat. A microscope slide (Table 1 item #11) was positioned diagonally under the section (Figure 5I). By lifting the slide, the section adhered to its surface (Figure 5J). To maintain the orientation of the sections, every slide was labeled with the corresponding location number and an x-z-coordinate system (Figure 5K). Sections were set to dry for at least 5 h at RT or 2 h at 37 °C and stored at 4 °C for a maximum of 10 days for histological staining and a maximum of 5 days before immunohistochemistry (IHC) or immunohistofluorescence (IHF). Figure 6 (and Supplementary Figures S1, S2) presents an overview of all structures found within the ML and visible in the sections: Former air-filled, now HistoCURE-filled PMP fibers, held in place by warp threads, and the deposits within the now HistoCURE-filled blood compartment. The x-z-coordinate system allows the definition of the blood flow direction downwards within the images.

### 3.7 Histological protocols

**Figure 7:**
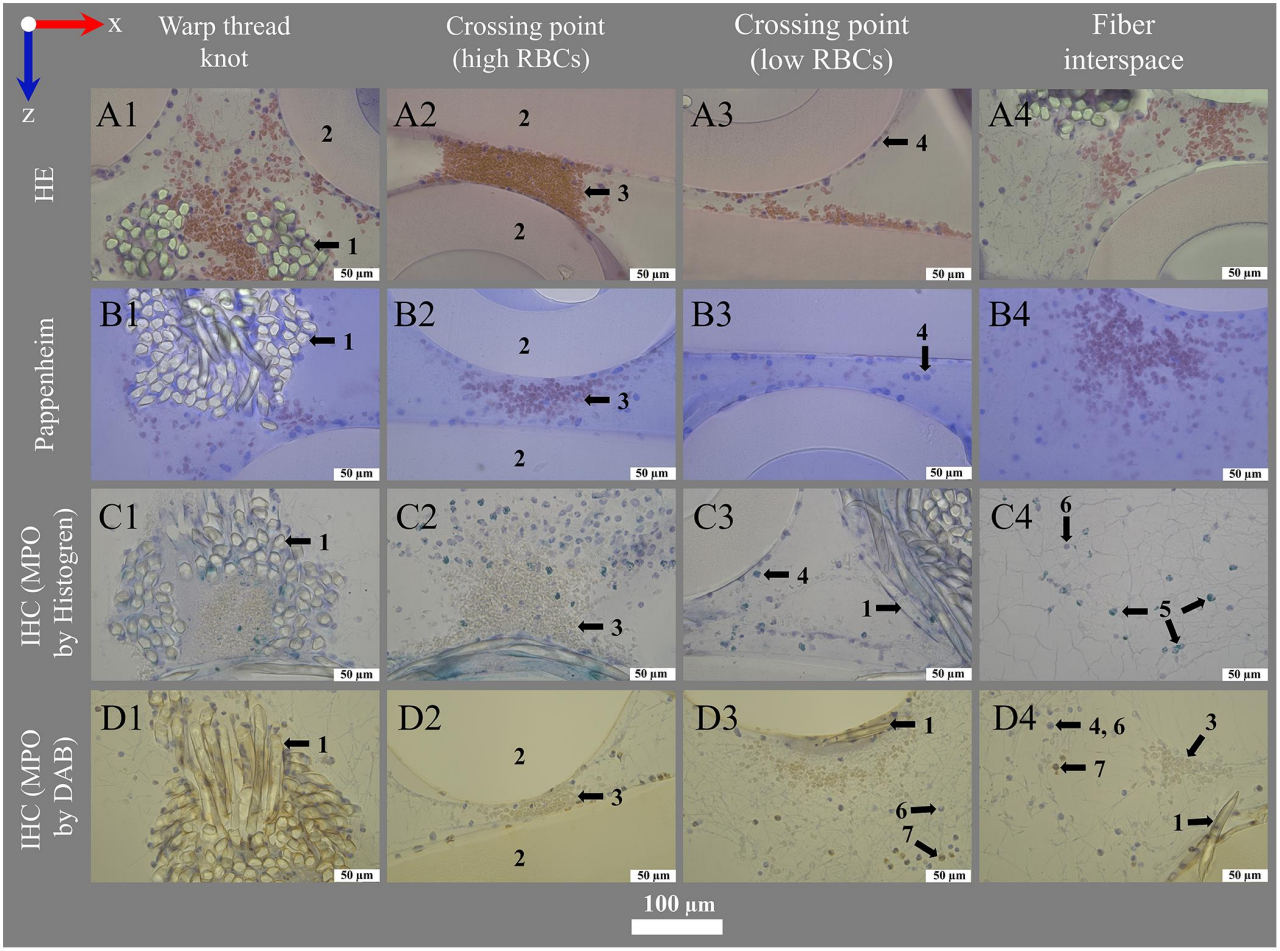
Histological and IHC staining for transmitting light microscopy. Images show (left to right) deposits within or around the warp thread, crossing points with high and low load of red blood cells (RBCs) and the interspace of the blood compartment; blood flow in z-direction. Optimal staining results were performed using (**A1–A4**) hematoxylin eosin staining (HE) with 1.0% eosin (RBCs pink-red) and 6 min hemalum (nucleated cells blue) incubation mounted with VectaMount®; (**B1–B4**) Pappenheim staining with 3 min May-Grunwald (nucleated cells blue) and Giemsa (RBCs pink) stainings mounted with Fluoromount-G™; IHC stainings using anti-MPO antibody at dilution 1:100 and visualization with (**C1–C4**) Histogreen (MPO green) or (**D1–D4**) DAB (MPO brown), both counterstained with hemalum (nucleated cells blue) and mounted with Entellan. The sections were slightly affected by the stains: **(A)** Eosin with VectaMount® AQ mounting led to a slight color change of PMP fibers to rose; **(A,C)** hemalum stained HistoCURE 8100 lightly gray-blue; **(B)** Pappenheim mounted with Fluoromount-G™ stained HistoCURE 8100 light blue and the PMP fibers light purple; **(D)** DAB stained all plastic components lightly yellow-brown.

**Figure 8:**
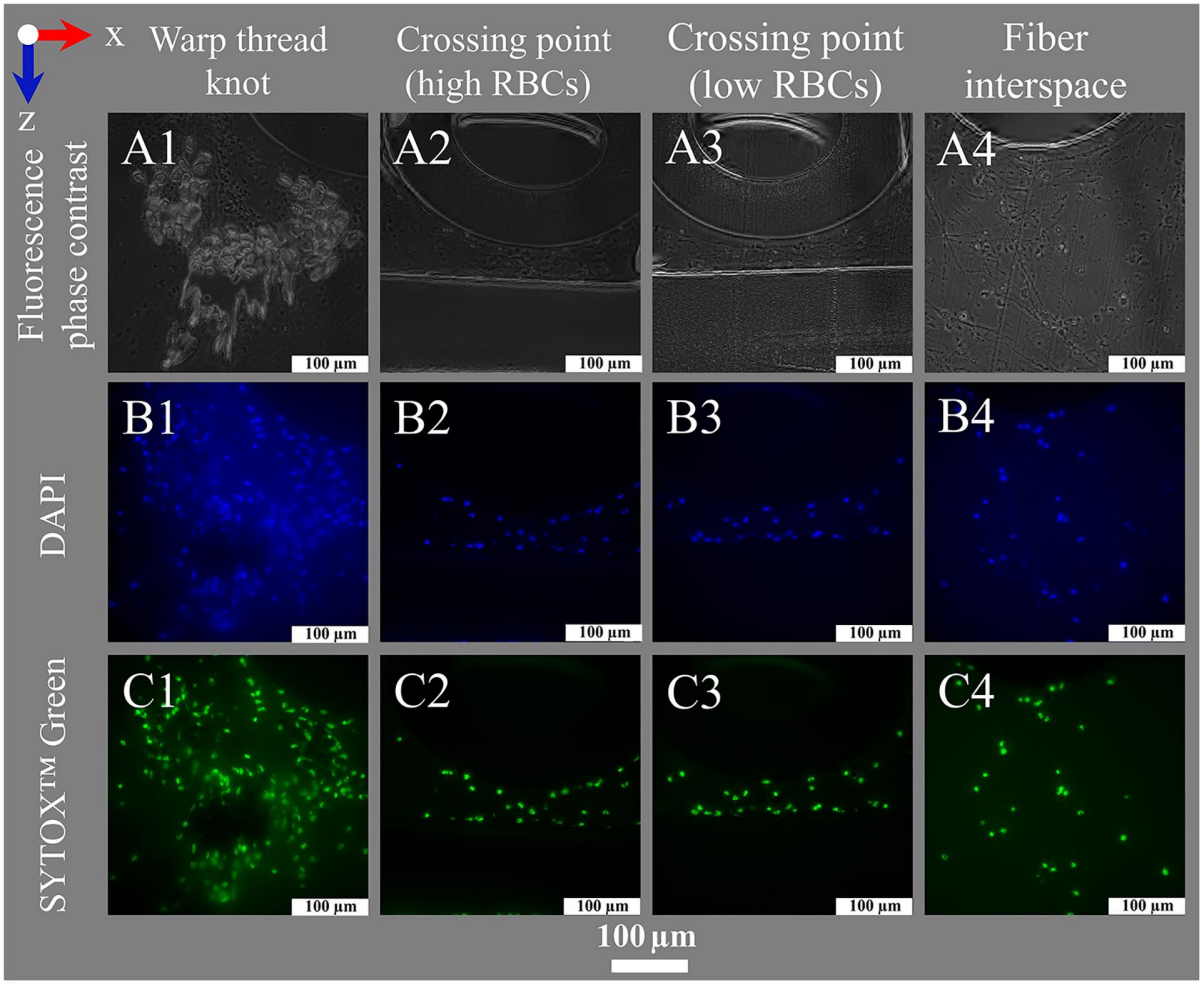
Nuclei staining for fluorescence microscopy. Images show (left to right) deposits within or around the warp thread, crossing points with high and low loads of RBCs and the interspace of the blood compartment; blood flow in z-direction. (**A1–A4**) Fluorescence phase contrast for structural information; Fluorescence staining results using (**B1–B4**) DAPI (DNA blue) and (**C1–C4**) SYTOX™ Green (DNA green).

**Figure 9:**
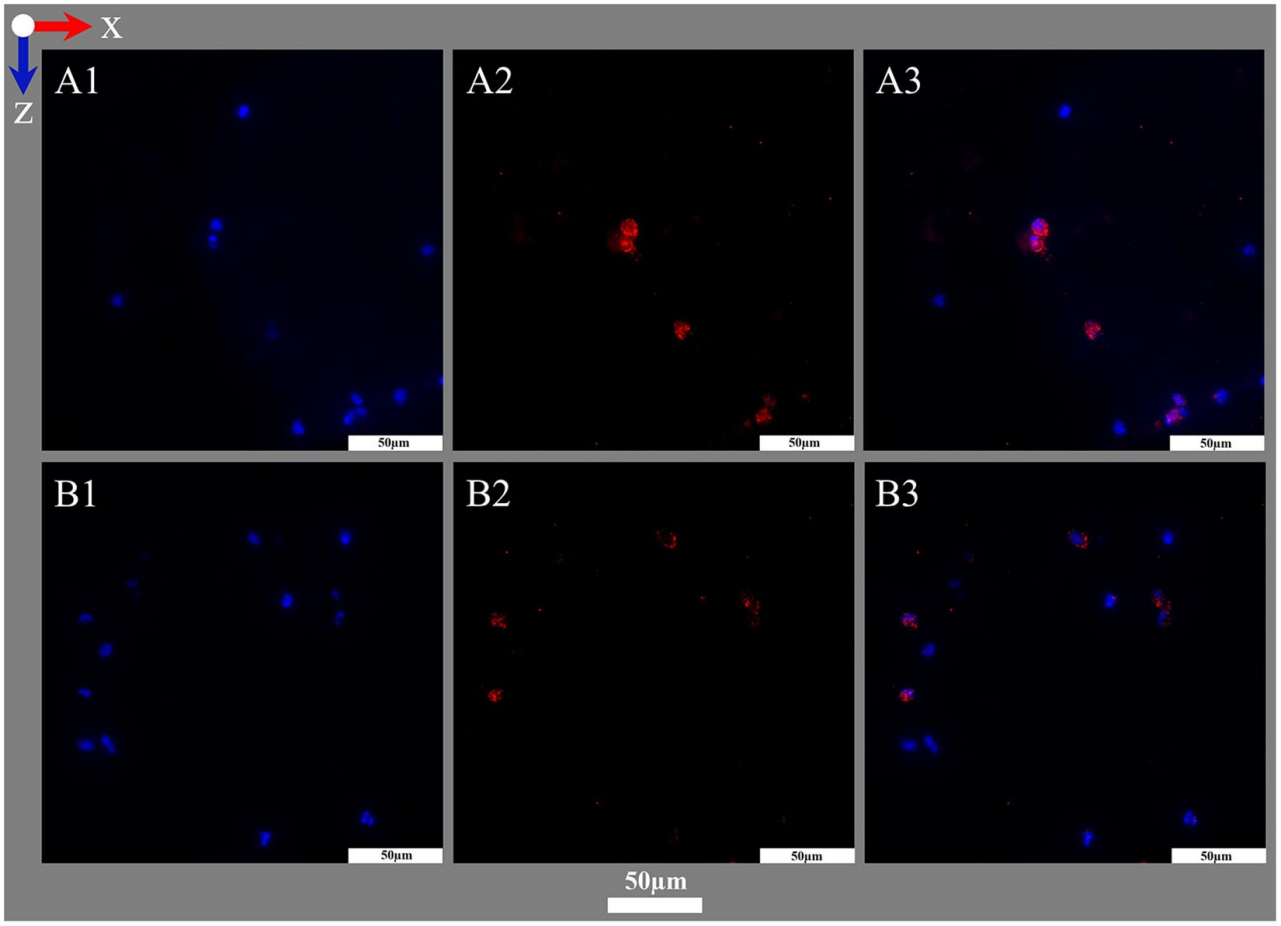
Visualization of cytoplasm with MPO using IHF. **(A,B)** Images acquired at two exemplary positions both acquired within the interspace of the blood compartment; blood flow in z-direction. (**A1,B1**) DAPI staining of nuclei (blue). (**A2,B2**) IHF staining against MPO (red). (**A3,B3**) Overlay images showing MPO staining closely associated with nuclei.

Staining procedures for embedded specimen have been developed by adoption of the state of the art protocols of our laboratory for paraffin embedded tissues and non-embedded MLs (20) under consideration of protocols from Malik et al. (33), De Jonge et al. (34), and Ponsioen et al. (35). The final protocols are presented in the following. Examples are depicted in Figure 7 (transmission light microscopy) and Figures 8, 9 (fluorescence microscopy). The protocols for polymer embedded samples are generally characterized by the use of higher staining concentrations. This is due to the fact that the polymer in the sample cannot be deplasticized and consequently fewer binding sites are available. Nevertheless, sufficient image and signal quality was possible.

#### 3.7.1 Positive and negative controls

To generate positive controls, paraffin embedded human tonsils were stained using the original protocol for paraffin embedded tissues. Further, HistoCURE 8100 embedded human tonsils were embedded and stained according to the protocols provided for the ML. The tonsils were provided by the Department of Pathology of the University Hospital Regensburg.

Negative controls for histological protocols were naïve embedded sections. Negative controls of all antibody stainings were performed by either adding (A) no primary or secondary antibodies, (B) only secondary antibody or (C) isotype control antibody (Table 2 item #41) at dilution matching the primary antibody concentration.

#### 3.7.2 Histological staining

This section presents the developed protocols for hematoxylin-eosin staining (HE) and Pappenheim staining. For HE staining, dried sections were equibrillized with deionized water (2 min, RT) and incubated with Mayer's hemalum solution (Table 2 item #35; 6 min, RT). They were quickly rinsed with deionized water (10 s, RT), 0.1% HCl (Table 2 item #53) in distilled water (10 s, RT), and again deionized water. To enable the color shift to blue, sections were incubated with warm tap water (2 × 3 min, 20 °C) and rinsed with deionized water. Thereafter, sections were stained with 1% Eosin (Table 2 item #36; 30 s, RT) in distilled water (acidification with 1 drop of acetic acid/100 mL, Table 2 item #54) and rinsed with deionized water. Sections were dewatered by dipping in ethanol (3% × 100%; Table 2 item #52) and xylene (2×; Table 2 item #55) before mounting with Entellan™ (Table 2 item #48) with a cover slide (polymerization 24 h, 4 °C). This protocol provided the best staining results with the least staining of the polymer background or fibers (Figure 7A).

Alternatively, sections were dewatered by dipping in ethanol (2% × 70%, 2% × 96% 2% × 100%) and xylene (2×) before mounting with Entellan™ or directly mounted with VectaMount® AQ (Table 2 item #49). Staining with 0.1% Eosin (30 s, RT) was tested, but resulted in non-satisfactory staining of red blood cells (RBCs) despite similar background staining.

For Pappenheim staining, air dried sections were incubated with freshly filtered May-Grunwald solution (Table 2 item #37; 3 min, RT) and rinsed with distilled water (3 min). Giemsa staining (Table 2 item #38) was carried out at 1:20 dilution in distilled water (15 min, RT). Finally, samples were rinsed with distilled water and mounted with Fluoromount-G™ (Table 2 item #50) and a cover slide (polymerization 24 h, 4 °C, Figure 7B).

Other methods for mounting the slides, like air drying with or without adding a coverslip (with a drop of distilled water) right before microscopy or direct mounting with VectaMount® AQ and a cover slide (polymerization 24 h, 4 °C) resulted in stronger background staining with overall inferior staining quality. A shorter incubation with May-Grunwald stain (1 min, RT) reduced background staining but resulted in non-satisfactory staining of nucleated cells despite similar background intensity.

#### 3.7.3 Immunohistochemical staining

An IHC staining protocol was established using an antibody against myeloperoxydase (MPO). Variations of the protocol during elaboration are listed below.

To prevent dispersion of the liquid staining solution, the dried sections were framed with a lipophilic marker and set to dry (30 min, RT). Then, they were equibrillized with deionized water (5 min, RT). Endogenous peroxydase was blocked using 3% H_2_O_2_ (Table 2 item #56) in deionized water (2 × 5 min, RT) [protocol of our laboratory, (33)]. Samples were washed with deionized water (2 × 5 min, RT) and 0.1 M tris buffered saline [TBS: 0.424% m/v Tris Base, 2.6% m/v Tris-HCl, 8.76% m/v NaCl (Table 2 items #27–29) in distilled water, pH 7.4; 5 min, RT] before antigen retrieval with 0.05% trypsin (Table 2 item #57) with 0.1% CaCl_2_ (Table 2 item #58) in 0.1 M Tris-HCl buffer (2.12% m/v Tris Base, 13% m/v Tris-HCl in distilled water, pH 7.8; Table 2 items #29–30; 30 min, 37 °C, humid compartment) (35). Sections were washed with TBS (3 × 5 min, RT) and incubated (30 min, RT, humid compartment) in blocking buffer [1% m/v bovine serum albumin (BSA) + 0.2% m/v gelantine of cold water fish skin, Table 2 items #31–32, in TBS] + 10% normal goat serum (NGS, Table 2 item #32). Sections were incubated with primary antibody against MPO (dilution 1:100; Table 2 item #39) or isotype control antibody (Table 2 item #41) in blocking buffer + 2% NGS (18 h, RT, humid compartment). Samples were washed with TBS (2 × 5 min, RT) and PBS (5 min, RT) before incubation with biotinylated secondary antibody (Table 2 item #42), diluted 1:100 in PBS + 2% NGS (1 h, RT, humid compartment). Sections were washed with PBS (3 × 5 min, RT) and primed with avidin biotin complex (ABC, Table 2 item #44; 1 h, RT, humid compartment) and again washed with PBS (3 × 5 min, RT). To visualize the bound secondary antibody, sections were incubated with A) Histogreen [pre-incubation with substrate + buffer (10 min, RT, humid compartment); incubation with substrate + buffer + H_2_O_2_ (3–4 min, RT, humid compartment), Table 2 item #45, Figure 7C] or B) DAB-Liquid [per-incubation with DAB + PBS (10 min, RT, humid compartment); incubation with DAB + buffer (3–4 min, RT, humid compartment), Table 2 item #46, Figure 7D]. Thereafter, sections were rinsed with deionized water (3× 5 min, RT) and counterstained with Mayer's hemalum for 30 s. Sections were rinsed with deionized water, warm tap water (20 °C) and again deionized water before dewatering by dipping in 100% ethanol (3×) and xylene (2×). Sections were mounted with Entellan™ and covered with a cover slide (polymerization 24 h, 4 °C).

During establishment, several variations of the protocol were tested: In contrast to 3% H_2_O_2,_ blocking of endogenous peroxydase with 0.06% H_2_O_2_ showed non-specific positivity in negative controls and was thus discarded. The primary MPO antibody was tested for 1:50, 1:300 and 1:600 dilutions, which all showed inferior staining results. Incubation temperatures of the primary antibody were varied to A) 4 °C for 18 h (20), with reduced staining intensity or B) first 1 h at 37 °C and 17 h at RT (34), which provided similar results compared to the final protocol. To evaluate the staining results, Mayer's hemalum counterstaining was performed by dipping (2×) during the establishment of the methods. Afterwards, counterstaining with Mayer's hemalum was tested for 5 s, 30 s, 60 s, 5 min and 10 min, but 30 s incubation provided the best results for visualization of nuclei (Mayer's hemalum, blue) combined with MPO (Histogreen, green or DAB, brown).

Paraffin and HistoCURE embedded human tonsils served as positive controls for the staining results. Briefly, paraffin was removed of the 5 µm sections by incubation with xylene (2 × 10 min, RT), 100% ethanol (2 × 5 min, RT), 96% ethanol (2 × 5 min, RT), 70% ethanol (1 × 5 min, RT) and deionized water (2 × 5 min, RT). Endogenous peroxydase was blocked by 3% H_2_O_2_ in deionized water (2 × 5 min, RT), then sections were washed with deionized water. For antigen retrieval, sections were incubated in citric buffer (pH 6, Table 2 item #59; dilution 1:10 in distilled water) in a water bath at 97.7 °C–98.4 °C (1 h) and washed with deionized water after a 20 min cooling period. The samples were dried, framed with a lipophilic marker, and set to dry (30 min, RT). Sections were equilibrated (deionized water, 2 × 5 min, RT), washed in PBS (3 × 5 min) and incubated in blocking buffer + 10% NGS (30 min, RT, humid compartment) before incubation with the primary anti-MPO antibody at dilution 1:600 in PBS + 5% NGS (18 h, 4 °C, humid compartment). Thereafter, samples were tempered (1 h, RT), washed with PBS (3× 5 min) and incubated with biotinylated secondary antibody at dilution 1:300 in PBS + 5% NGS (1 h, RT, humid compartment). Sections were then washed in PBS (3 × 5 min), incubated with ABC (1 h, RT, humid compartment) and washed again in PBS (3 × 5 min). Visualization of the bound antibody was performed with Histogreen or DAB-Liquid [pre-incubation (10 min, RT); incubation (3–4 min, RT)]. As soon as staining was visible, the incubation was stopped by rinsing with deionized water (3 × 5 min, RT). For counterstaining, samples were dipped (2x) in Mayer's hemalum at dilution 1:2 with distilled water, then rinsed with deionized water, warm tap water (20 °C) and again deionized water. For dewatering, samples were dipped in 100% ethanol (3×) and xylene (2×). Sections were mounted with Entellan™ under a cover slide (polymerization 24 h, 4 °C).

#### 3.7.4 Fluorescence and immunofluorescence staining

Protocols for fluorescence staining with 4′,6-diamidino-2-phenylindole (DAPI), SYTOX™ Green as well as IHF using an antibody against MPO and vWF were established. Variations of the protocol during elaboration are listed below. As soon as fluorescent agents were applied, sections were handled under protection from daylight.

The fluorescent DNA stains DAPI and SYTOX™ Green were used to visualize nucleated cells. Therefore, dried sections, framed with a lipophilic marker were equilibrated in deionized water (5 min, RT) and stained with SYTOX™ Green (Table 2 item #47) at dilution 1:500 in TBS (30 min, RT). They were then washed with TBS (3 × 5 min, RT) and mounted with Flouromount-G™ DAPI (Table 2 item #51) with a cover slide (polymerization 24 h, 4 °C; Figure 8).

During protocol establishment, antigen retrieval with 0.05% trypsin and 0.1% CaCl_2_ in Tris-HCl buffer (30 min, 37 °C, humid compartment) (35) prior SYTOX™ Green application showed no noticeable effect in the results. SYTOX™ Green was tested at dilutions 1:500, 1:750, 1:1,000 in TBS (30 min, RT), but 1:500 showed the best results.

For IHF, dried sections, framed with a lipophilic marker were equilibrated in deionized water (5 min, RT). Antigen retrieval was performed with 0.05% trypsin and 0.1% CaCl_2_ in Tris-HCl buffer (2.12% m/v Tris Base, 13% m/v Tris-HCl in distilled water, pH 7.8; 30 min, 37 °C, humid compartment) (35). The sections were washed with TBS (3 × 5 min, RT) and incubated with blocking buffer [1% m/v bovine serum albumin (BSA), 0.2% m/v gelantine of cold water fish skin in TBS] + 10% normal donkey serum (NDS, Table 2 item #34; 30 min, RT, humid compartment). Then, sections were incubated with primary antibody against MPO (dilution 1:100) or vWF (dilution 1:100, Table 2 item #40) or isotype control antibody in blocking buffer + 2% NDS (18 h, RT, humid compartment). Samples were washed with TBS (3 × 5 min, RT) before incubation with a fluorophore conjugated secondary antibody (Table 2 item #43) at dilution 1:500 in TBS + 2% NDS (1 h, RT, humid compartment). Finally, sections were washed with TBS (3 × 5 min, RT), mounted with Flouromount-G™ DAPI to counterstain nucleated cells and covered with a cover slide (polymerization 24 h, 4 °C; Figure 9).

### 3.8 Microscopic imaging

Imaging was conducted with a DMi8 microscope with a LED 5 light source (Table 1 item #12) equipped with a K3C RGB camera (Table 1 item #13) for transmitting light microscopy and a K5 grayscale camera (Table 1 item #14) with a bandpass and a channel specific filter (Table 1 items #15–16, bandpass filter: 420–450 nm, 506–532 nm, 578–610 nm, 666–724 nm; channel specific filter: 420–460 nm, 500–540 nm, 565–615 nm, 662–738 nm) for fluorescence microscopy. The microscope was controlled using LAS X software (Table 1 item #17) and was housed in a dark enclosure to prevent the fluorophore from fading. At 400-fold magnification it was visible that the sections were slightly uneven. To compensate for the limited depth of field and obtain sharp images, a series of consecutive images at different z-positions (z-stack images) were taken. Each z-stack was then combined by the LAS X software into a sharp image (extended depth of field).

Images were taken in 25-fold magnification to get an overview of the sectioning quality and arrangement of the hollow-fibers. For more detailed information about the staining results, images were taken in 100- and 400-fold magnification. For 100-fold magnification, z-stacks were acquired until 45 µm of z-size with a z-step size of 3.80 µm (system optimized). For 400-fold magnification, z-size ranged between 5 and 35 µm with a z-step size of 0.29 µm (system optimized). Plain areas of the section used rather small z-size (5–20 µm) whereas the visualization of warp threads required higher z-size (up to 35 µm). Detailed descriptions of the procedure for transmitting light and fluorescence microscopy are presented in the following sections. Post-processing of images was performed using Adobe Photoshop CS5 (Table 1 item #18) if necessary (to reduce background).

#### 3.8.1 Transmitting light microscopy

For transmitting light microscopy (3:2 rectangular images: *x* = 3072 pixels, *y* = 2048 pixels), one channel was used, and the colors were visualized in the split RGB channel (color mode). The range of pixel values was used throughout (0–4,095). In all magnifications, fluorescence intensity manager (FIM) was used and the camera gained 100% of the light. Exposure time was set to 85 ms (gain: 1). For 25-fold magnification the light intensity was at 50% (aperture: 3, light-field: 26). For 100-fold magnification the light intensity was at 30% (aperture: 10, light-field: 37). For 400-fold magnification, the light intensity was at 50% (aperture: 21, light-field: 28).

#### 3.8.2 Fluorescence microscopy

In fluorescence microscopy (squared images: x = 2,048 pixels), images were obtained individually at an illumination wavelength corresponding to the excitation spectrum of the respective fluorophore. DAPI was visualized in blue at an excitation wavelength of 390 nm (58% LED intensity, filter: 420–460 nm) and an exposure time of 250 ms. For SYTOX™ Green, the fluorescein isothiocyanate (fitc)-channel (visualization in green) was excited at 475 nm (30% LED intensity, filter: 500–540 nm) with 10 ms exposure time. MPO was visualized in red in the far red (fr)-channel at an excitation wavelength of 635 nm (58% LED intensity, filter: 662–738 nm) with 800 ms exposure time. For all three channels, the FIM was used at 100% with an illumination field set to 6 and all light was sent to the camera (100%). To gain more information regarding the co-localization of stained biological deposits and different structures within the ML (hollow-fibers, warp threads), fluorescence phase contrast was added (excitation at 390 nm, 58% LED intensity, filter transmission 100%, exposure time: 25 ms, color settings for visualization in overlay images: RGB 0–50–80). DAPI and SYTOX™ Green channels were visualized using the automated grayscale adjustment. For IHF, the fr-channel gray values were set to 350–1,200 for visualization of MPO and vWF. An overlay image of all used channels was created. Further raw images using the full grayscale (65,535 gray values) were exported.

## 4 Results

**Figure 10:**
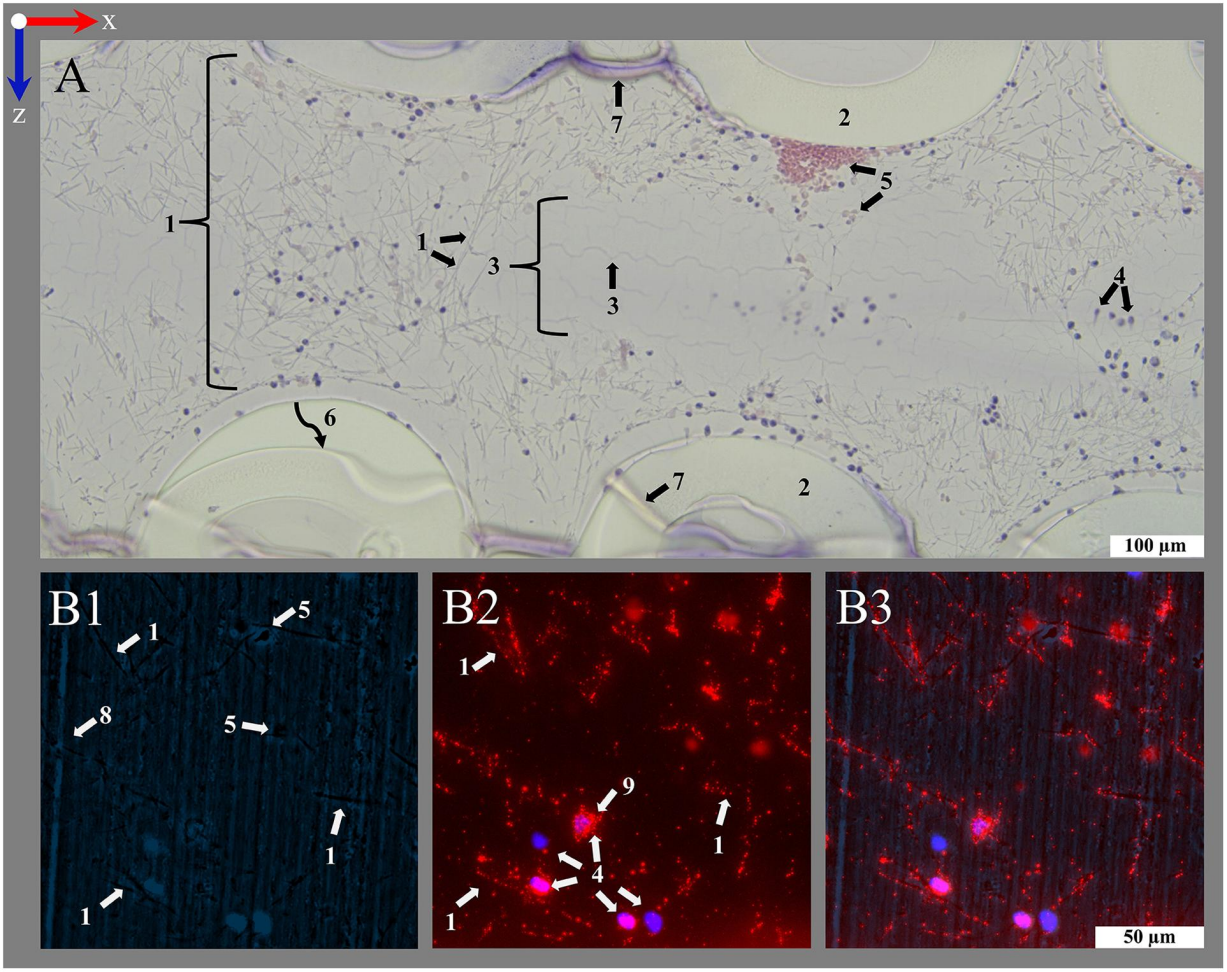
Identification of different biological clot components. Differentiation between (1) biological cobweb structures spanning between the (2) gas exchange fibers and (3) geometric cracks within the HistoCURE 8100 visible in cobweb-free areas of the blood compartment; blood flow in z-direction. **(A)** Staining with HE for differentiation of (4) nucleated cells (blue), (5) RBCs (pink) and fibrin fibers in the cobweb (light purple). Cellular deposits were either found on the surface of gas exchange fibers or within the cobweb, but not in cobweb-free areas. (6) The dislocation of the gas exchange fibers during microtome cutting did not influence the location of deposits fixed in place by HistoCURE 8100; (7) artifact folds. **(B)** Identification of vWF using IHF. (**B1**) Fluorescence phase contrast marking surface structures (1) cobweb structures and (8) sectioning artifact. (**B2**) IHF of nucleated cells (DAPI, blue) and vWF (red). VWF was either (1) spaced out as beaded dots along a strand or (9) located closely to nucleated cells as granula. (**B3**) Overlay image showing vWF strands aligned congruently with cobweb structures from the phase contrast image.

The present work describes a protocol for polymer embedding of MLs using a combination of the polymer resins HistoCURE 8100 and Technovit® 3040. The embedded fiber mats were mostly held securely in the polymer scaffold and did not collapse when cut with the microtome. In some cases, single dislocated hollow-fibers could be observed (Figure 10A) during sectioning. However, the rigid polymer scaffold allowed not only sectioning of the hollow-fibers but also the multi-layered biological deposits which were safely embedded within and kept in native position. An elongation of the PMP hollow-fibers (as detected in Technovit® 9100) or the formation of air bubbles within the polymer was not detected. Sections were possible until 5 µm of thickness (Figure 6A). Because of better handling and more plain sections, a thickness of 10 µm (Figures 6B–D, 7–10) was considered ideal. HistoCURE 8100 demonstrated the ability to safely fix biological deposits both in visually clotted (Figure 6A) as well as clot-free (Figure 6B) areas. This enabled the observation of large deposits that adhered to the hollow-fibers surface and formed structures spanning from one fiber to another. Clots in the warp thread were also fixed in place (Figure 6C). Both PMP fibers and the warp thread were accessible for longitudinal and cross-sectioning (Figures 6C,D).

Using histological stainings (HE, Pappenheim, Mayer's Hemalum, Figures 6, 7, 10A), the high prevalence of leukocytes and RBCs, which were mainly attached to the fiber surface, within the warp thread or entangled in fiber mat spanning multidimensional cobwebs could be visualized. A further differentiation of cells and proteins within the biological deposits was achieved by IHC (Figures 7C,D) and IHF stainings (Figures 8, 9, 10B). As demonstrated in Figure 10A, sometimes thin geometric cracks within the HistoCURE 8100 were visible but were clearly distinguishable from biological cobwebs. These cracks were the result of an interaction between the polymer and solvents required before mounting.

It was observed that vWF structures existed either spaced out as beaded dots or located closely to nucleated cells as granula. In the overlay image the beaded dots were congruent to cobweb structures derived from fluorescence phase contrast (Figure 10B3). Biological cobweb structures are three-dimensional which means that strands were only accessible for IHF if they were exactly aligned within the sectioning plane. Strands that are crossing the sectioning plane were therefore only visible as dots in IHF, whereas the cobweb within the 10 µm section is visible with fluorescence phase contrast (Figure 10B1) as well as with transmitting light microscopy (Figure 10A). Therefore, we propose vWF as a main component of the cobweb structures (Figure 10B).

So far, the warp threads were not accessible by top view imaging due to their three-dimensional structure, hindering access to the relatively high cell loads, intertwined between the multiple strands of the threads (Figure 1). Embedding the ML with HistoCURE 8100 allowed the sectioning of warp threads as cross-sections (Figure 6C) or in longitudinal direction (Figure 6D). Histological staining and IHC allowed a deeper insight into the cells adherent to the warp thread (Figures 6C1–2, 6D1–2). The signals of deposits in fluorescent staining and IHF were limited by light refraction on the transparent strands of the warp thread (Figures 6C3–4, 6D3–4). While the determination of fluorescent areas might give insight into the density of cellular deposits within the warp thread, cell differentiation by fluorescent staining and IHF might be more suitable in other areas but the strands of the warp thread.

**Figure 11:**
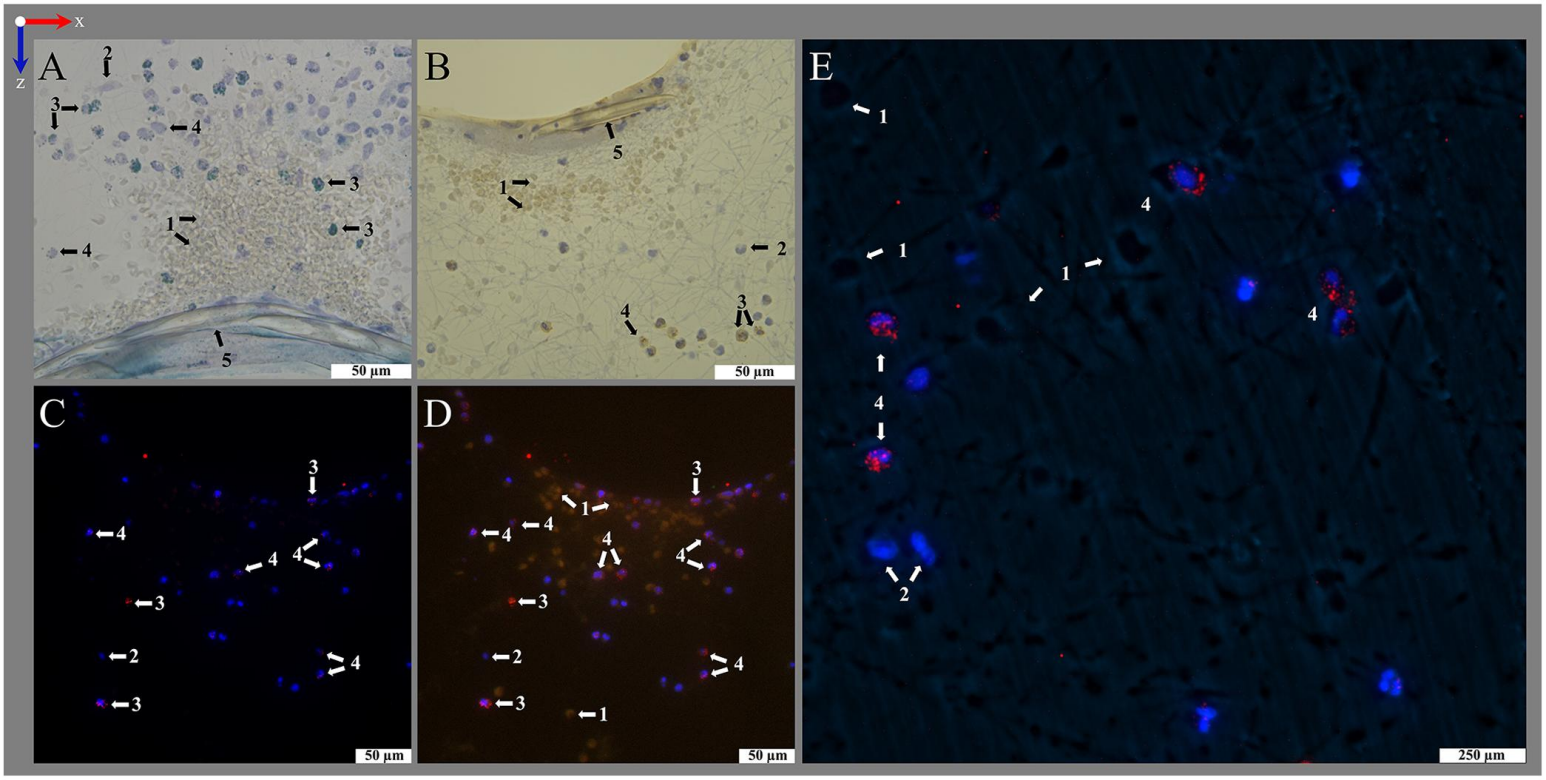
Differentiation of MPO load in IHC and IHF staining. IHC stainings with anti-MPO stained with **(A)** Histogreen (MPO green) and **(B)** DAB (MPO brown) and counterstaining with hemalum (nuclei blue); blood flow in z-direction. Cellular deposits mainly consisted of RBCs (1, non-stained) and nucleated cells (hemalum, blue) without (2) and with (3–4) MPO expression, mostly found as granular structures. Differentiation between cells with (3) large amounts of MPO antigen and (4) smaller amounts of MPO antigen was possible in both staining methods, but Histogreen visualization presented more clear results. The cells displayed are located close to (5) a warp thread. **(C–E)** IHF presented another method to visualize even small amounts of MPO (red) close to DAPI-stained nuclei (blue) and offers easier automated detection methods based on split channels. Therefore, visualization of MPO was tested in tritc- and fr-channel during protocol development. **(C,D)** Overlay images of MPO in the tritc-channel with different gray value thresholds combined with DAPI. The gray values used for the tritc-channel (MPO) and fitc-channel (empty control) were **(C)** 150–250 and **(D)** 100–250. Small granular MPO was more visible if the threshold was set to lower gray values. However, this also visualized autofluorescent RBCs (1). RBC autofluorescence was eliminated at gray values above 150 in fitc- and tritc-channel. **(E)** Overlay image of fluorescence phase contrast combined with MPO in fr-channel and DAPI. Application of protocol's secondary antibody with excitation in the fr-channel reduces the detection of RBC autofluorescence. RBC silhouettes were then only visible using fluorescence phase contrast (1).

IHC provided a relatively easy, resistant (as not sensitive to daylight unlike IHF) and reproducible method for the detection of biological proteins. Both Histogreen and DAB staining allowed a graduation of MPO load of nucleated cells (Figures 11A,B). Yet, the main advantage of antibody staining using IHC with Histogreen was the well visible differentiation between strongly MPO-positive cells and cells with only small granular MPO (light green).

### 4.1 Trouble shooting

Pre-infiltration is necessary to achieve a sufficient binding of the HistoCURE 8100 with the clots. The lower viscous pre-infiltration solution is soaked by the clots allowing a hardening within. Without pre-infiltration the clots were only encapsulated by the polymer but not stabilized from the inside. This resulted in inhomogeneous hardness of the specimens which did not allow microtome sectioning. Pre-infiltration is therefore especially important for MLs with high clot burden.

Despite HistoCURE 8100 did not allow deplastification, in histological and fluorescence staining, all deposits within the sections were accessible due to small molecule size of the dyes. Yet, only the surface of the section was accessible for antibody staining (IHC and IHF). Thus, the amount of accessible antigen on the surface might be low (e.g., if it is located in the cytoplasm like MPO and the nucleus is cut through in this section, Figures 11A,B) despite a larger amount of it just a little deeper below the surface. Therefore, the antibody dilution had to be increased compared to paraffin sections during establishment of antibody stainings on HistoCURE 8100.

The visualization of the structures in different fluorescence channels by using fluorescent staining and IHF was developed successfully. While nuclei were apparent in the DAPI-channel, MPO and vWF were visualized in red. This allowed the investigation of both structures separately and their colocalization in merged images (Figures 9, 10B).

**Figure 12:**
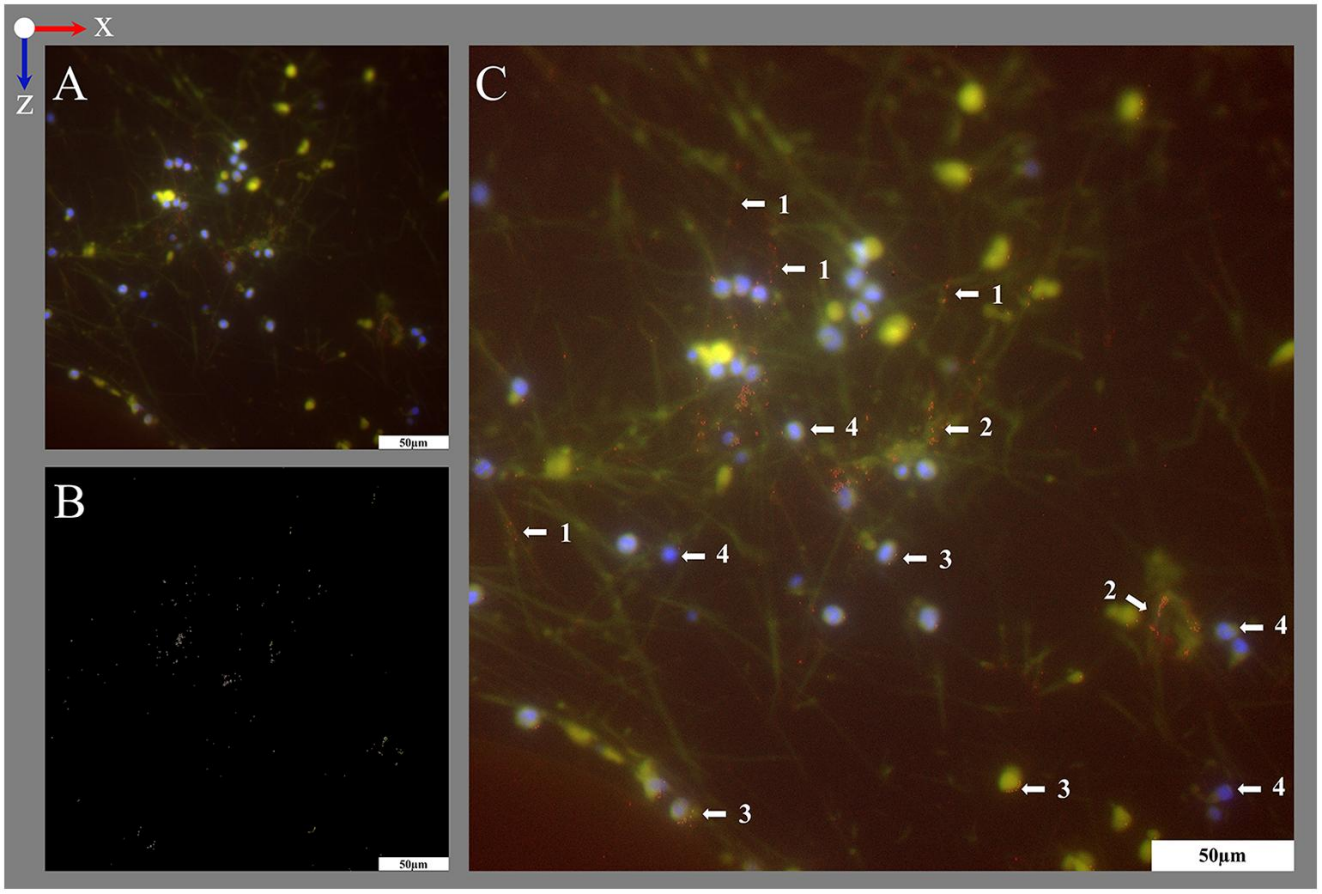
Visualization of vWF structures using the tritc-channel and digital image subtraction. The image was acquired within the interspace of the blood compartment; blood flow in z-direction. **(A)** Overlay image of vWF (red) in the tritc-channel combined with DAPI stained nuclei (blue). Since vWF structures were accessible for staining only on the surface of the sections, they only yielded low signal and required a low gray value threshold. There, the signal of autofluorescent structures that were located beneath the surface of the 10 µm thick section interfered with the vWF signal. Therefore, the fitc-channel (empty control) was acquired for detection of autofluorescence only (autofluorescence yellow in overlay). Gray value thresholds for fitc- and tritc-channel were set to 100–250 to allow the detection of small vWF structures. **(B)** To map out the vWF signal, digital gray scale images subtraction of the tritc- and the fitc-channel was conducted. **(C)** Enhanced overlay image with the vWF signal (**B**, visualized in red) added to the original overlay image **(A)**. This allows the location of the vWF signal on top of the autofluorescent structures within the section. The vWF was detected on (1) strand-like and on (2) accumulated autofluorescent structures and (3) close to (4) DAPI-stained nuclei. Split channel images are provided in Supplementary Figure S3.

During protocol development, MPO and vWF were visualized using the state of the art secondary antibody (Donkey, anti rabbit AlexaFluor 594, 711-585-152, Dianova, Hamburg, Germany) in the tetramethylrhodamine isothiocyanate (tritc)-channel according to Wagner et al. (20) at an excitation wavelength of 560 nm (58% LED intensity, filter: 565–615 nm) with 60 ms exposure time (Figures 11C,D, 12A). To suppress autofluorescence signal, the tritc-channel gray values were set to 150–250, yet small granula were visible best using 100–250. Yet, non-stained RBCs within the 10 μm thick section, which were visible in fluorescence phase contrast, were autofluorescent in fitc-and tritc-channels when gray values were set to 100–250 (Figure 11D). The autofluorescent RBCs appeared as cloud-like structures with not clearly defined margins. When assembled in clots, it appeared as an area of autofluorescent material. Yet, due to the brilliant staining qualities of the MPO antibody staining (access only on the surface of the section) in IHF, the gray values of MPO were above even larger RBC clots and the granular structure of MPO could be differentiated clearly. The autofluorescence of RBCs was suppressed in visualizations at gray values of 150–250, yet reduced the visibility of small MPO granula (Figure 11C). By introducing the secondary antibody in the fr-channel presented in this protocol, the detection of autofluorescence signal was significantly reduced (Figure 11E). The silhouettes of structures within the section were then only visible in fluorescence phase contrast (Figure 11E).

Similarly, when visualizing vWF in tritc-channel, relatively short exposure times were chosen to minimize autofluorescence (Figure 12A; Supplementary Figure S3). However, the vWF signal was of low brilliance. Therefore, an empty control image was acquired in the fitc-channel which was subsequently subtracted from the tritc-channel image. This digital grayscale image subtraction yielded the isolated vWF signal (Figure 12B). An enhanced overlay image was generated after adding the isolated vWF signal to the original overlay image (Figure 12C). Still, the use of the protocol's secondary antibody in the fr-channel improved the signal quality (Figure 10B).

We observed the unspecific accumulation of antibody staining sometimes occurred in deep microtome knife artifacts resulting from the high material hardness of the polymer. This was significantly reduced by frequently changing the knife's position. Minor knife artifacts, however, did not show any antibody accumulation (Figure 10B1).

## 5 Discussion

This protocol represents a controllable and reproducible procedure for polymer embedding of an entire clinically used ML. The protocol fulfills the aimed requirements of a significant increase in mechanical stability of both the ML interior and the cellular and extracellular deposits, enabling histological examinations of the cut sections.

### 5.1 Significance of methodology

The use of ECMO increases worldwide as treatment option for severe respiratory and cardiocirculatory failure (4, 36–38). As coagulation-associated technical complications represent a major limitation of ECMO (7, 22, 39–41) and are not yet sufficiently understood to ensure effective prediction and treatment, further research is required, particularly with regard to clot formation in the ML.

Polymer embedding of MLs is an innovative method for the preservation of sensitive clot structures for histological examinations in areas that could not be assessed previously. Following embedding, deposits around the entire circumference of the hollow-fibers, within the warp threads, and structures spanning across fiber mats can be examined in native position without visible manipulation. Furthermore, the stabilization of the clot structures is sufficient to allow reliable cross-sections and histological staining. Preliminary results show a clear superiority over conventional fiber mat staining with top view fluorescence microscopy, as only visually clot-free areas on single fiber mats could be examined (19, 20). This methodology represents a promising approach for the investigation of intra-device clot formation. It considerably expands the histological examination methods and could make an important contribution to a deeper understanding of the complex, multifactorial clotting pathways in MLs.

Moreover, a transfer to other applications in medicine is conceivable, as the method essentially describes the embedding of large-volume samples (400 mL) in polymer to be stained and examined histologically. Studies on other blood-carrying medical devices such as ECMO blood pumps or dialysis filters might represent potential applications for adapted embedding and staining protocols.

### 5.2 Quality of embedding and specimen extraction

After embedding, the ML was solid enough to perform microtome sections with a thickness of 5–10 µm without any optical artifacts. Section thickness of 10 µm was considered optimal for handling. Microtome sections present membrane morphologies and can be stained with common histological, IHC, and IHF staining. The warp thread surrounding single hollow-fibers remains intact and histological examinations of thread deposits can be carried out. Our protocol allows the investigation of immunological processes around hollow-fibers which are not possible with conventional paraffin embedding: Embedding of large specimens consisting of various materials such as MLs in original state, localization of clots (by MDCT) and targeting them for further investigation, and reproducible serial sections of hollow-fibers and deposits. For this reason, polymer embedding appears to be a promising method that will be foundation for new insights in the complex field of intra-device clot formation.

### 5.3 Histological staining

Using polymer embedding for MLs allows reproducible serial cross-sections of ML hollow-fibers and biological material in clots and clot-free regions. Further, examination of deposits in the direction of blood flow is feasible, which was a missing dimension in previous studies on top-view ML hollow-fibers (19, 20). This might allow conclusions to be drawn between flow dynamics and clotting mechanisms. Thin serial sections enable the investigation of different components of the coagulation cascade through (immuno-)histological staining. An approximation of three-dimensional structures within the ML and their investigation in the direction of blood flow is possible.

Staining of polymer-embedded samples with both standard histological and IHC or IHF stains presents a major challenge due to the material properties of many polymers. After evaluating various methods, protocols for standard histological staining (HE, Pappenheim staining) as well as IHC and IHF protocols for DNA staining (DAPI and SYTOX™ Green) and antibody staining for MPO and vWF were established. These were assessed and adjusted using both positive and negative controls with satisfactory and reproducible results. Depending on the specific requirements, the protocols may be further adapted and extended to include other antibodies. Furthermore, the use of additional fluorophores, particularly in the long-wave red spectrum, e.g., in the near-infrared range, could extend the depth of this protocol. However, our currently available light source (LED 5) does not feature this wavelength.

### 5.4 Alternative embedding approaches

Finding the right embedding polymer was a challenge. To make the work easier for other research groups, we would like to share our experience on other tested plastics that are not part of the final protocol. Technovit® 2000 LC with curing agent was tested in advance. This showed great stability and easy processing. However, this is only suitable for light microscopy. Histological staining was not possible. Technovit® 9100 was also tested due to the possibility of performing IHC tests. Deplastification of the polymer after microtome cutting is possible according to the manufacturer. An interaction between the Technovit® 9100 base solution and the hollow-fibers was detected. However, this resulted in an elongation of the hollow-fibers by approx. 10%. In addition, the xylene recommended by the manufacturer for preparation proved to be not suitable for this certain application. Dissolution of the plasticizers by xylene caused the ML housing to become brittle. Furthermore, xylene dissolved the coloring of the ML housing, resulting in a blue coloration of the hollow-fibers.

### 5.5 Limitations

This method shows promising results for the PMP gas exchange hollow-fibers. However, the heat exchange hollow-fibers consist of TPU which become softer and more easily deformed. In preliminary tests without pre-infiltration a deformation of the TPU fibers was also detectable. An examination of TPU hollow-fibers is still possible but a dislocation of the hollow-fibers must be considered. HistoCURE 8100 does not allow a deplastification of the polymer (32), which is why staining can only be carried out with the polymer in place. Consequently, only the two-dimensional surface of the microtome sections was accessible for antibody staining while histological staining dyes reached more into the depth of the section. Yet, this protocol allows thin microtome sections. An approximation of a large three-dimensional specimen can be performed by investigating a high number of very thin two-dimensional slices.

## 6 Conclusion

In conclusion, the presented methodology is a controllable and reliable approach to increase the mechanical stability of ML hollow-fibers and biological deposits. Through this, histological investigation of previously not-evaluable areas within MLs, especially clots and deposits spreading in the direction of the blood flow, can now be conducted. This provides access to new scientific findings and ultimately understand the complex and multifactorial clotting pathways in ECMO MLs.

## Data Availability

The original contributions presented in the study are included in the article/Supplementary Material, further inquiries can be directed to the corresponding author.
