# Supplementary material for: Polymer embedding of membrane lungs for histological investigations of intra-device clot formation
Source: Front Cardiovasc Med. 2026 Feb 4;13:1650978. doi: 10.3389/fcvm.2026.1650978 (PMC12913521; doi:10.3389/fcvm.2026.1650978)
Supplement: Supplementary file 1 [file Supplementaryfile1.pdf]

frontiers

Supplementary Material

Polymer embedding of membrane lungs for histological investigations of intra-device clot formation

Michael Kranz, Maria Stella Wagner, Daniel Pointner, Moritz Haus, Matthias Lubnow, Karla Lehle, and Lars Krenkel*

***Correspondence: Lars Krenkel, lars.krenkel@oth-regensburg.de**

1 Supplementary Figures

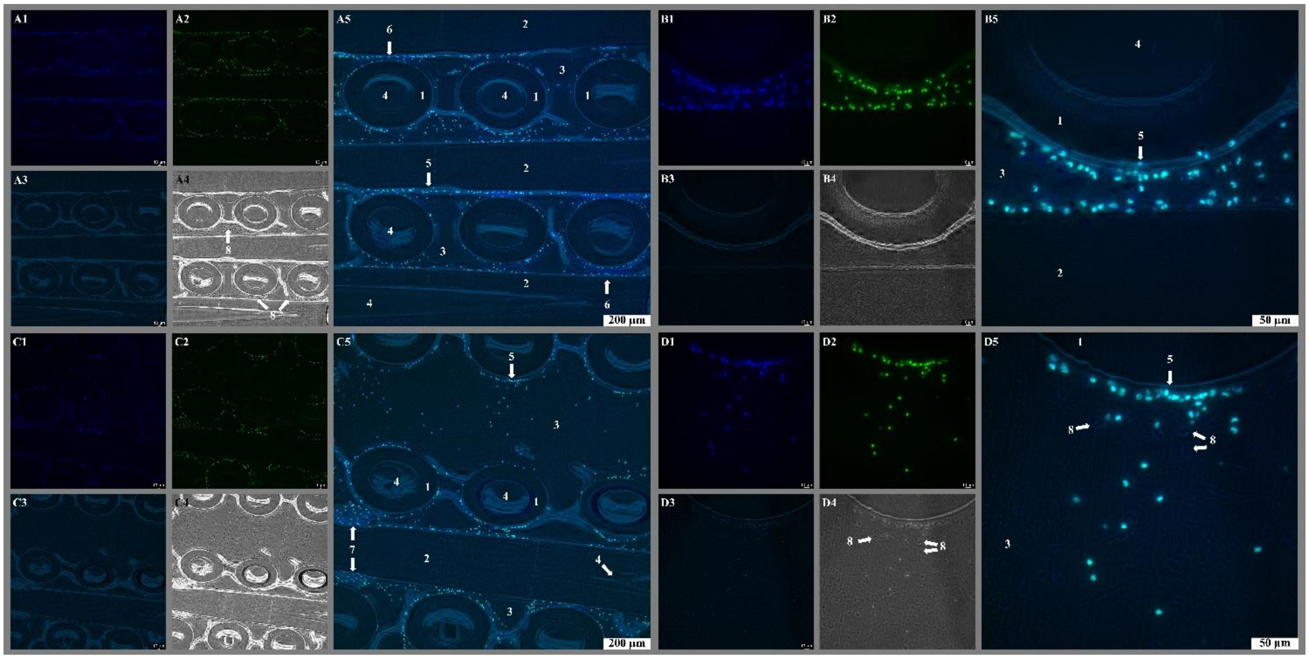

Supplementary Figure S1: Fluorescence microscopic images of embedded ML specimens of clotted and clot-free specimen. Sections originating from a (**A,B**) clotted and (**C,D**) clot-free region of the ML. (**A,C**) Overview of multiple (1) cross and (2) longitudinal sections of PMP fibers. The (3) blood compartment as well as (4) the inside of the fibers are filled with HistoCURE 8100. Especially the (5) the surface of PMP fibers, (6) crossing points of two PMP fibers, and (7) the warp thread are prone to attachment of deposits. (**B,D**) Detailed images allow a closer look at the deposits along the surface of the fibers (B) particularly in close proximity of a crossing point or (D) stretching in the interspace between the fibers. Biological deposits in the blood compartment are stained with (**A1-D1**) DAPI and (**A2-D2**) SYTOX™ Green for visualization of nuclei (DAPI blue; SYTOX™ Green green). Fluorescence phase contrast image for visualization of hollow-fibers and warp threads in (**A3-D3**) turquoise and (**A4-D4**) for better visualization of RBCs (8, black) in gray-scale. (**A5-D5**) Overlay of the images of the first three images allow co-localization of structures.

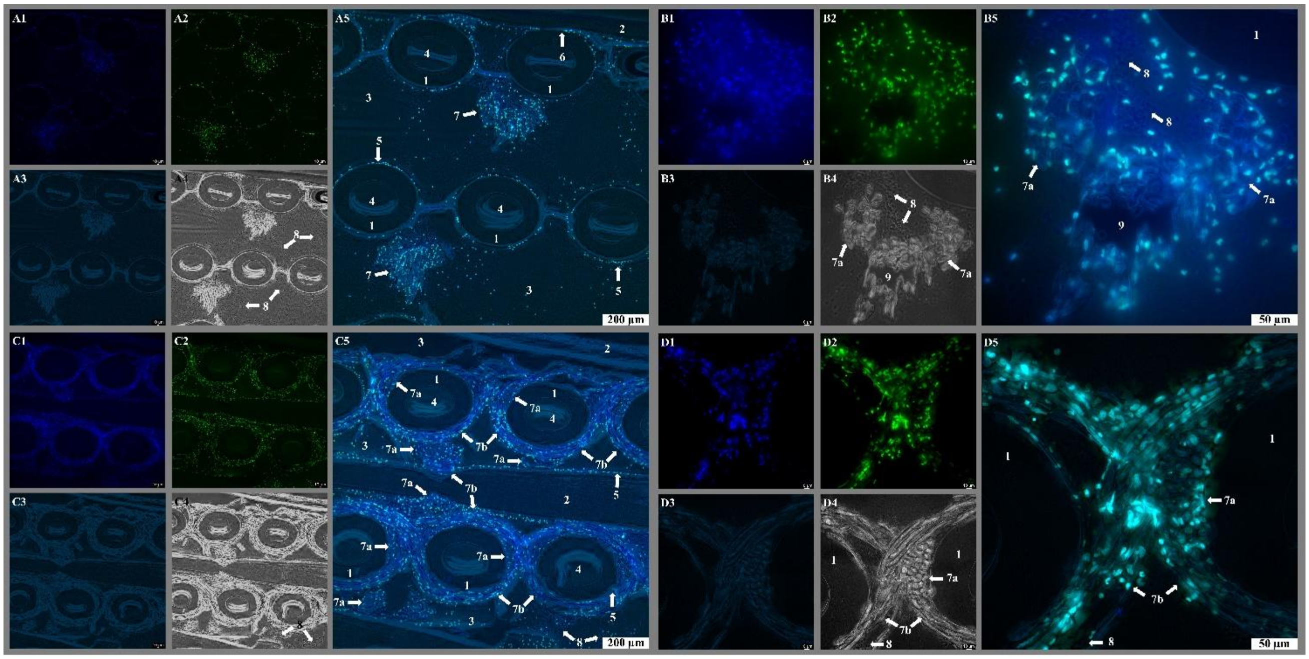

Supplementary Figure S2: Fluorescence microscopic images of embedded ML specimens with warp threads. Sections including (A,B) a cross-section of a warp thread knot and (C,D) a longitudinal cut of the warp thread. (A,C) Overview of multiple (1) cross and (2) longitudinal sections of polymethylpentene (PMP) fibers. The (3) blood compartment as well as (4) the inside of the fibers are filled with HistoCURE 8100. Especially the (5) the surface of PMP fibers, (6) crossing points of two PMP fibers, and (7) the warp thread ((7a) cross/ (7b) longitudinal section of strands) are prone to attachment of deposits. (B,D) Detailed images allow a closer look at the deposits within (B) a knot of the warp thread and (D) between the strands of the longitudinally cut warp thread. Biological deposits in the blood compartment are stained with (A1-D1) DAPI and (A2-D2) SYTOX™ Green for visualization of nuclei (DAPI blue; SYTOX™ Green green). Fluorescence phase contrast image for visualization of hollow-fibers and warp threads in (A3-D3) turquoise and (A4-D4) for better visualization of RBCs (8, black) in gray-scale. (A5-D5) Overlay of the images of the first three images allow co-localization of structures ((9) artefact hole after microtome sectioning)

.

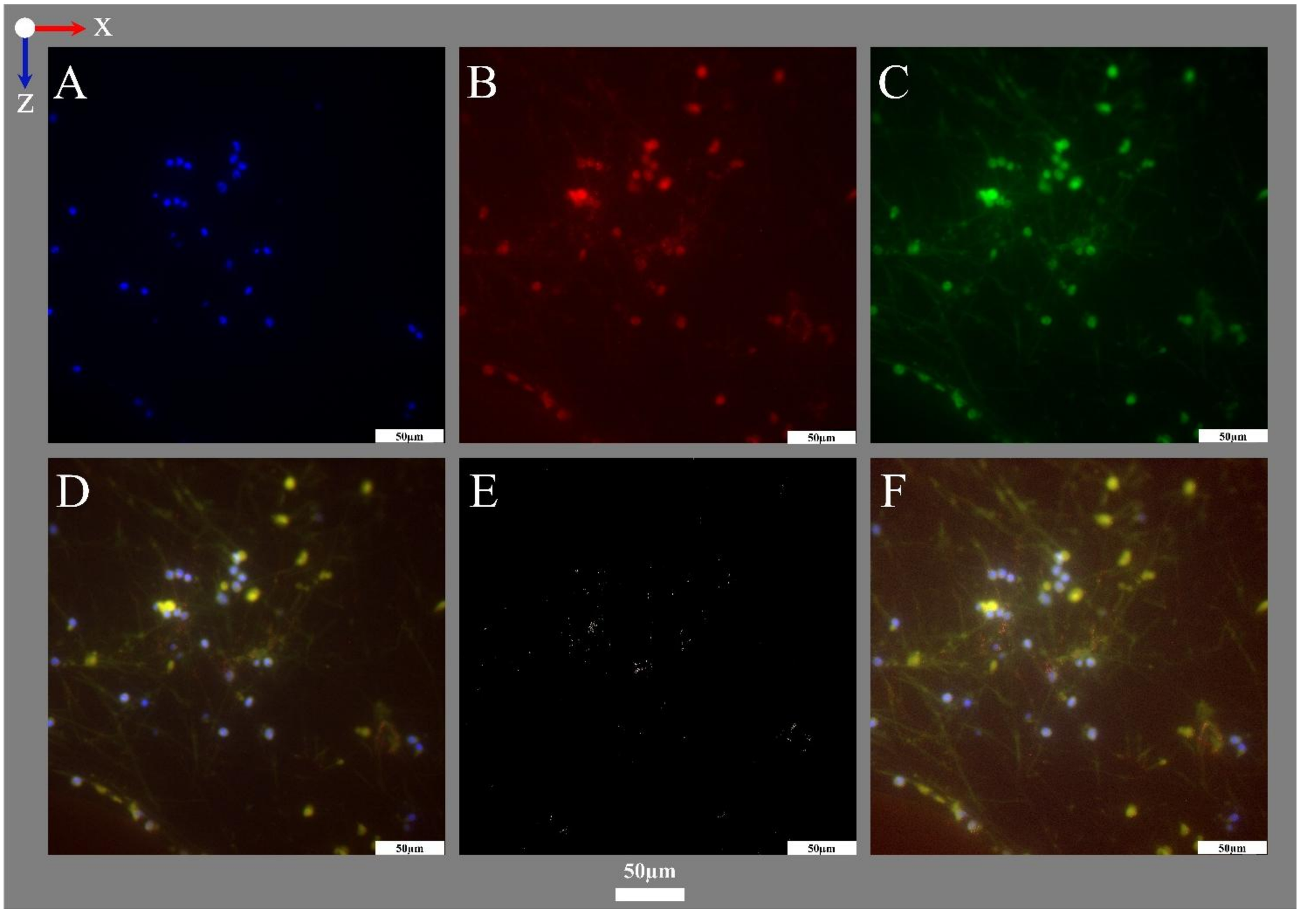

Supplementary Figure S3: Detailed images for digital image subtraction for vWF visualization. (A) DAPI-stained nuclei (blue), (B) tritc-channel image (vWF and autofluorescent structures visualized in red), (C) fitc-channel image (empty control, autofluorescent structures visualized in green) within the interspace of the blood compartment; blood flow in z-direction. (D) Overlay of (A-C) showing vWF in red and autofluorescence in yellow. (E) Digital subtraction image of (B) and (C) for detection of small vWF structures. (F) Enhanced overlay image with vWF signal (E) added to the original overlay image (D).
