# Supplementary material for: Polymer embedding of membrane lungs for histological investigations of intra-device clot formation
Source: Front Cardiovasc Med. 2026 Feb 4;13:1650978. doi: 10.3389/fcvm.2026.1650978 (PMC12913521; doi:10.3389/fcvm.2026.1650978)
Supplement: Supplementary file 2 [file Supplementaryfile2.pdf]

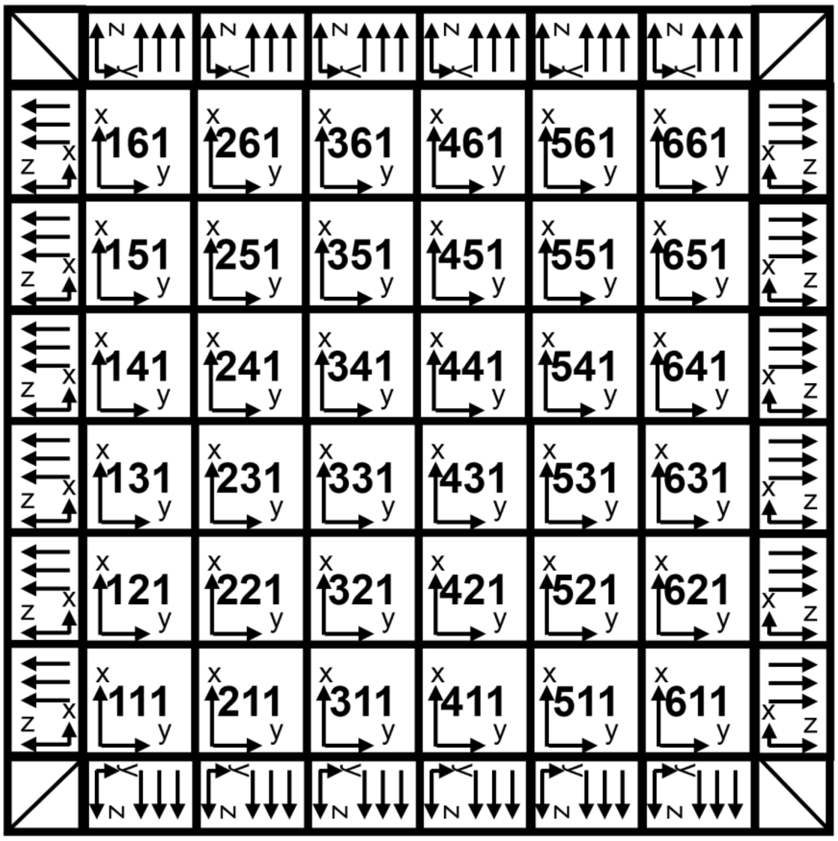
161 261 361 461 561 661
151 251 351 451 551 651
141 241 341 441 541 641
131 231 331 431 531 631
121 221 321 421 521 621
111 211 311 411 511 611

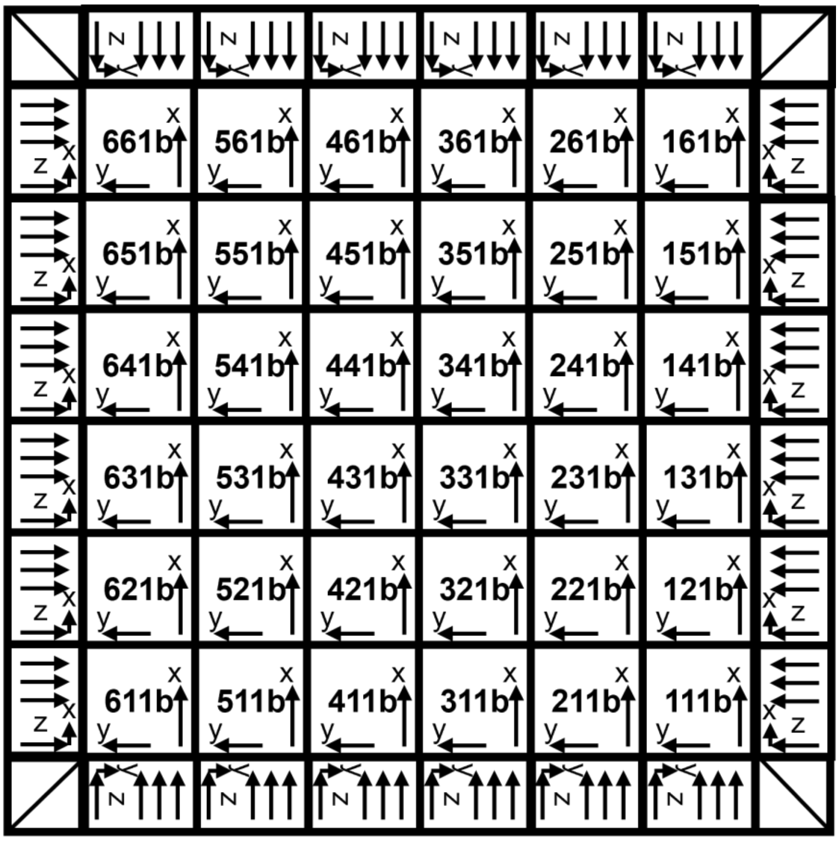
661b 561b 461b 361b 261b 161b
651b 551b 451b 351b 251b 151b
641b 541b 441b 341b 241b 141b
631b 531b 431b 331b 231b 131b
621b 521b 421b 321b 221b 121b
611b 511b 411b 311b 211b 111b

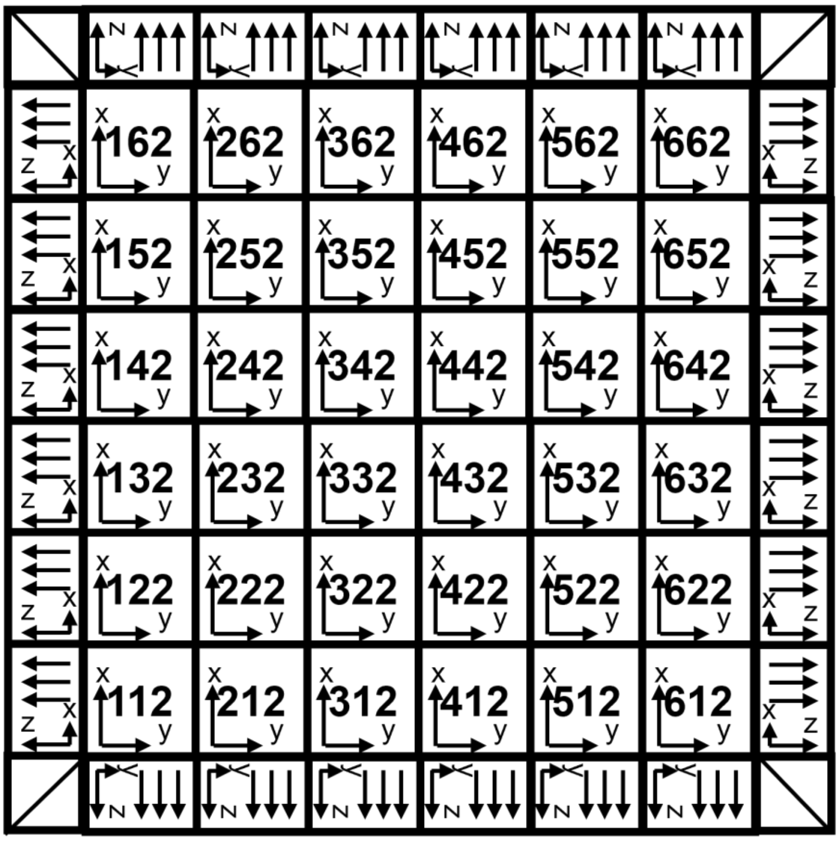
162 262 362 462 562 662
152 252 352 452 552 652
142 242 342 442 542 642
132 232 332 432 532 632
122 222 322 422 522 622
112 212 312 412 512 612
x y z

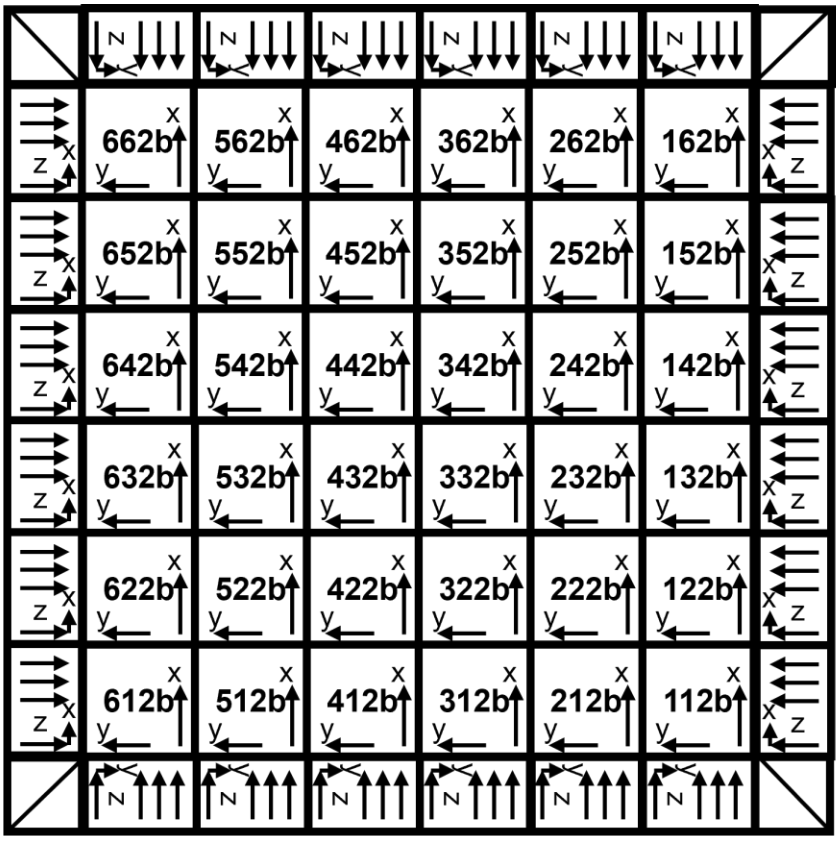
662b 562b 462b 362b 262b 162b
652b 552b 452b 352b 252b 152b
642b 542b 442b 342b 242b 142b
632b 532b 432b 332b 232b 132b
622b 522b 422b 322b 222b 122b
612b 512b 412b 312b 212b 112b
x y z

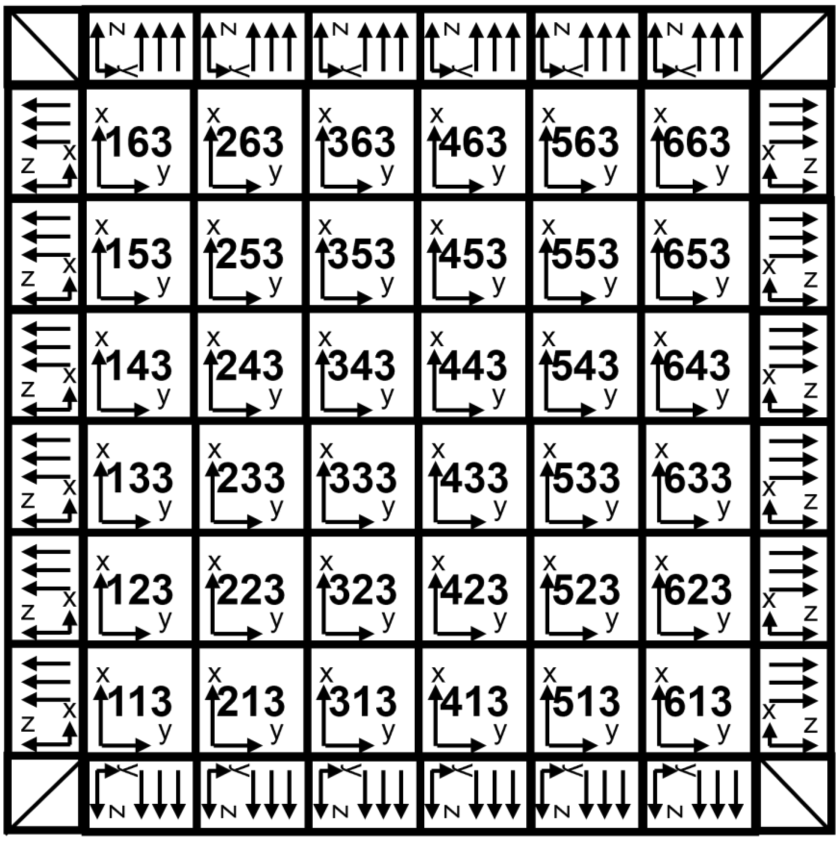
163 263 363 463 563 663
153 253 353 453 553 653
143 243 343 443 543 643
133 233 333 433 533 633
123 223 323 423 523 623
113 213 313 413 513 613
x y z

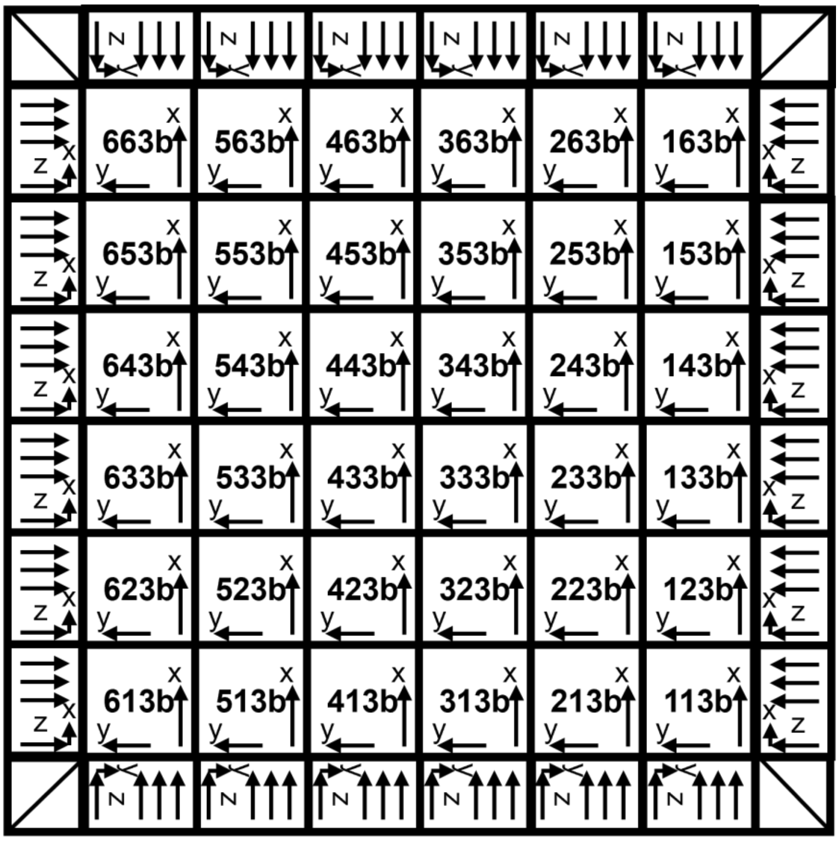
663b 563b 463b 363b 263b 163b
653b 553b 453b 353b 253b 153b
643b 543b 443b 343b 243b 143b
633b 533b 433b 333b 233b 133b
623b 523b 423b 323b 223b 123b
613b 513b 413b 313b 213b 113b
x y z

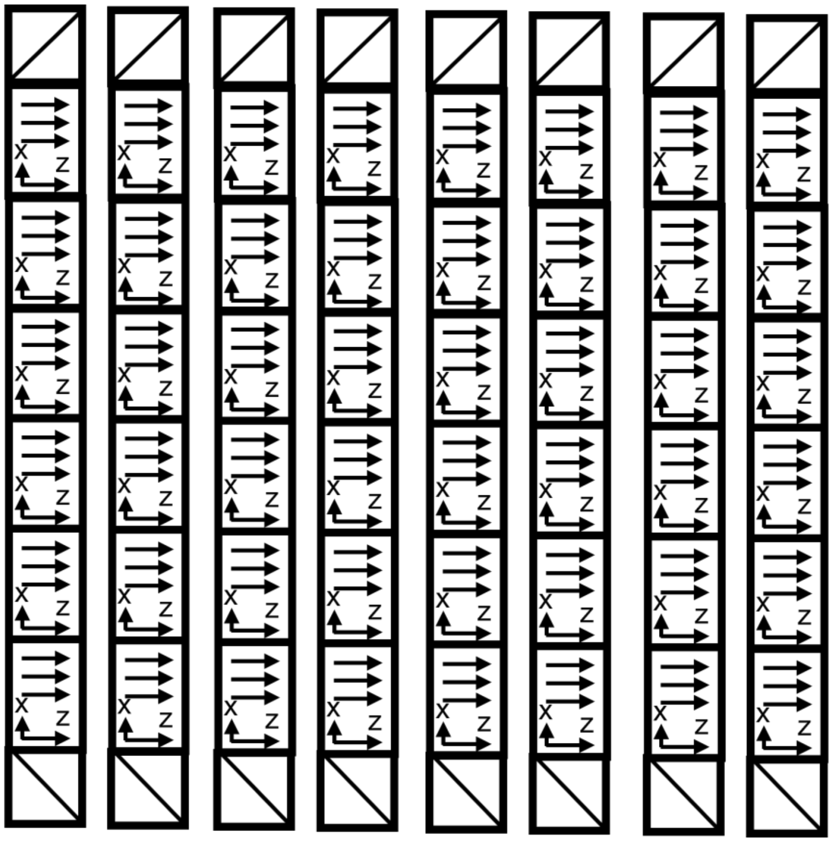
x
z

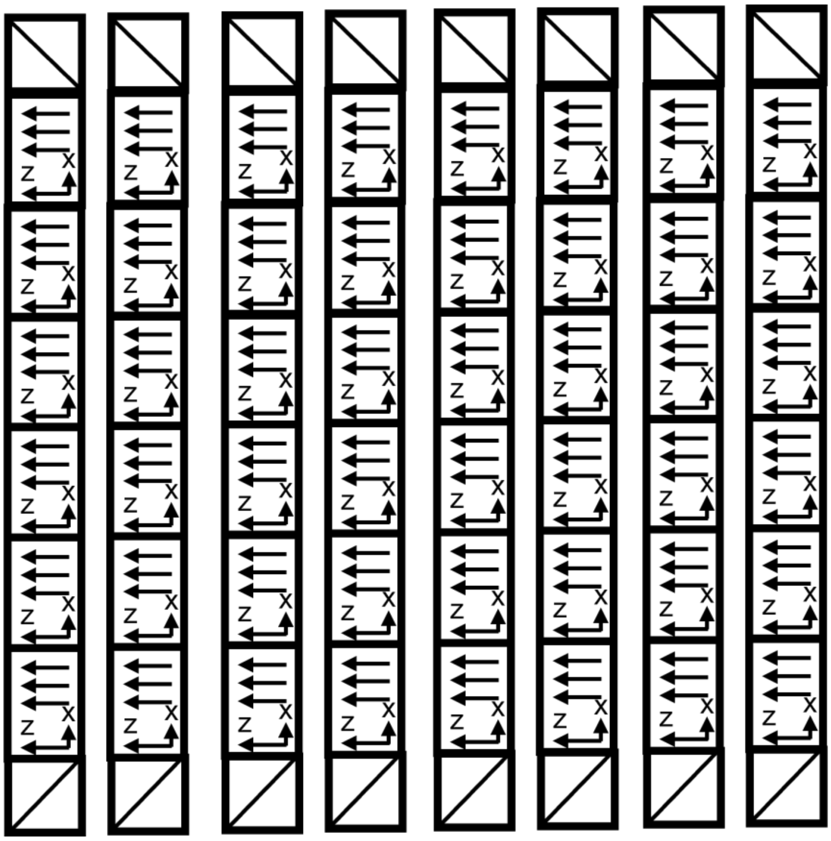
z
x
