# Supplementary figures and images for: Polymer embedding of membrane lungs for histological investigations of intra-device clot formation
Source: Front Cardiovasc Med. 2026 Feb 4;13:1650978. doi: 10.3389/fcvm.2026.1650978 (PMC12913521; doi:10.3389/fcvm.2026.1650978)

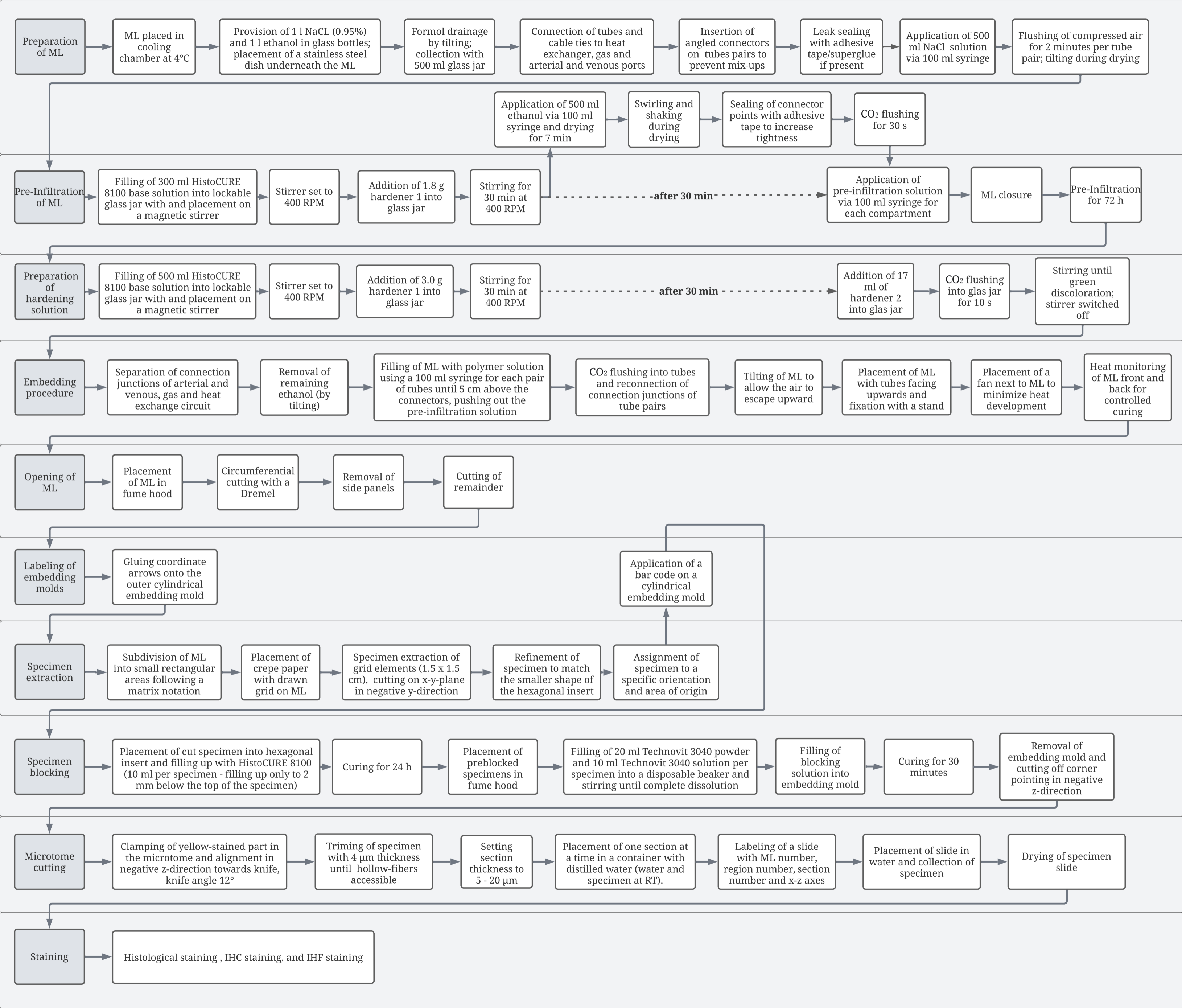

Supplement: Supplementary file 3 [file Supplementaryfile3.pdf]
