# Supplementary material for: Polymer embedding of membrane lungs for histological investigations of intra-device clot formation
Source: Front Cardiovasc Med. 2026 Feb 4;13:1650978. doi: 10.3389/fcvm.2026.1650978 (PMC12913521; doi:10.3389/fcvm.2026.1650978)

Labels for outside of specimen blocks and embedding molds

FLOW→z	→x	→y	FLOW→z	→x	→y	FLOW→z	→x	→y	FLOW→z	→x	→y
FLOW→z	→x	→y	FLOW→z	→x	→y	FLOW→z	→x	→y	FLOW→z	→x	→y
FLOW→z	→x	→y	FLOW→z	→x	→y	FLOW→z	→x	→y	FLOW→z	→x	→y
FLOW→z	→x	→y	FLOW→z	→x	→y	FLOW→z	→x	→y	FLOW→z	→x	→y
FLOW→z	→x	→y	FLOW→z	→x	→y	FLOW→z	→x	→y	FLOW→z	→x	→y
FLOW→z	→x	→y	FLOW→z	→x	→y	FLOW→z	→x	→y	FLOW→z	→x	→y
FLOW→z	→x	→y	FLOW→z	→x	→y	FLOW→z	→x	→y	FLOW→z	→x	→y
FLOW→z	→x	→y	FLOW→z	→x	→y	FLOW→z	→x	→y	FLOW→z	→x	→y
FLOW→z	→x	→y	FLOW→z	→x	→y	FLOW→z	→x	→y	FLOW→z	→x	→y
FLOW→z	→x	→y	FLOW→z	→x	→y	FLOW→z	→x	→y	FLOW→z	→x	→y
FLOW→z	→x	→y	FLOW→z	→x	→y	FLOW→z	→x	→y	FLOW→z	→x	→y
FLOW→z	→x	→y	FLOW→z	→x	→y	FLOW→z	→x	→y	FLOW→z	→x	→y
FLOW→z	→x	→y	FLOW→z	→x	→y	FLOW→z	→x	→y	FLOW→z	→x	→y
FLOW→z	→x	→y	FLOW→z	→x	→y	FLOW→z	→x	→y	FLOW→z	→x	→y
FLOW→z	→x	→y	FLOW→z	→x	→y	FLOW→z	→x	→y	FLOW→z	→x	→y
FLOW→z	→x	→y	FLOW→z	→x	→y	FLOW→z	→x	→y	FLOW→z	→x	→y
FLOW→z	→x	→y	FLOW→z	→x	→y	FLOW→z	→x	→y	FLOW→z	→x	→y
FLOW→z	→x	→y	FLOW→z	→x	→y	FLOW→z	→x	→y	FLOW→z	→x	→y
FLOW→z	→x	→y	FLOW→z	→x	→y	FLOW→z	→x	→y	FLOW→z	→x	→y
FLOW→z	→x	→y	FLOW→z	→x	→y	FLOW→z	→x	→y	FLOW→z	→x	→y
FLOW→z	→x	→y	FLOW→z	→x	→y	FLOW→z	→x	→y	FLOW→z	→x	→y
FLOW→z	→x	→y	FLOW→z	→x	→y	FLOW→z	→x	→y	FLOW→z	→x	→y
FLOW→z	→x	→y	FLOW→z	→x	→y	FLOW→z	→x	→y	FLOW→z	→x	→y
FLOW→z	→x	→y	FLOW→z	→x	→y	FLOW→z	→x	→y	FLOW→z	→x	→y
FLOW→z	→x	→y	FLOW→z	→x	→y	FLOW→z	→x	→y	FLOW→z	→x	→y
FLOW→z	→x	→y	FLOW→z	→x	→y	FLOW→z	→x	→y	FLOW→z	→x	→y
FLOW→z	→x	→y	FLOW→z	→x	→y	FLOW→z	→x	→y	FLOW→z	→x	→y
FLOW→z	→x	→y	FLOW→z	→x	→y	FLOW→z	→x	→y	FLOW→z	→x	→y

Labels especially for bottom of specimen blocks and bottom of molds

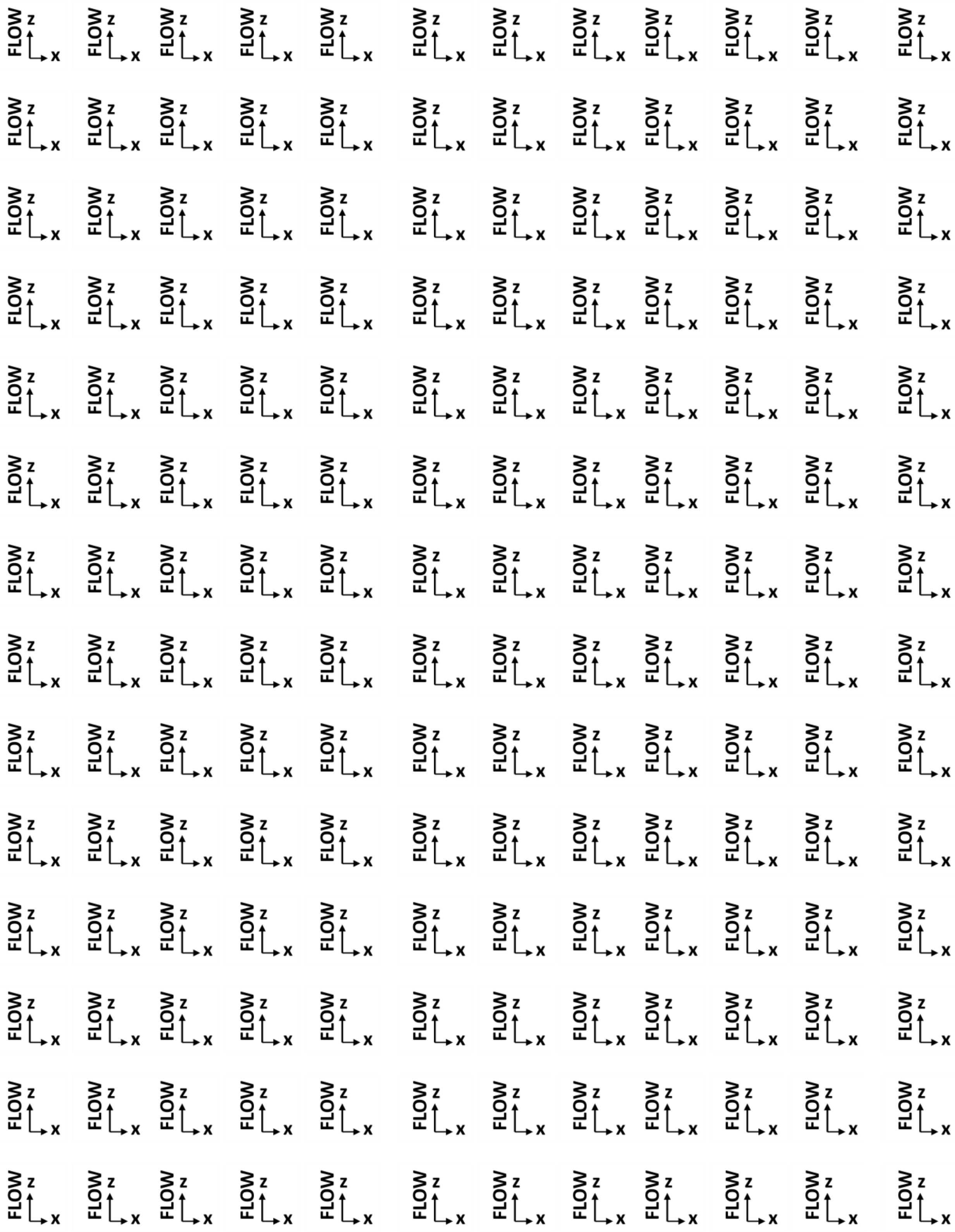

Supplement: Supplementary file 4 [file Supplementaryfile4.pdf]
